# Temperature-induced embryonic diapause in chickens is mediated by PKC-NF-κB-IRF1 signaling

**DOI:** 10.1186/s12915-023-01550-0

**Published:** 2023-03-08

**Authors:** Junxiao Ren, Zhengzheng Hu, Quanlin Li, Shuang Gu, Fangren Lan, Xiqiong Wang, Jianbo Li, Junying Li, Liwa Shao, Ning Yang, Congjiao Sun

**Affiliations:** Department of Animal Genetics and Breeding, College of Animal Science and Technology, China Agricultural University, Beijing, 100193 China

**Keywords:** Diapause, Embryo, Cell proliferation, Cold stress, PKC-NF-κB signaling

## Abstract

**Background:**

Embryonic diapause (dormancy) is a state of temporary arrest of embryonic development that is triggered by unfavorable conditions and serves as an evolutionary strategy to ensure reproductive survival. Unlike maternally-controlled embryonic diapause in mammals, chicken embryonic diapause is critically dependent on the environmental temperature. However, the molecular control of diapause in avian species remains largely uncharacterized. In this study, we evaluated the dynamic transcriptomic and phosphoproteomic profiles of chicken embryos in pre-diapause, diapause, and reactivated states.

**Results:**

Our data demonstrated a characteristic gene expression pattern in effects on cell survival-associated and stress response signaling pathways. Unlike mammalian diapause, mTOR signaling is not responsible for chicken diapause. However, cold stress responsive genes, such as IRF1, were identified as key regulators of diapause. Further *in vitro* investigation showed that cold stress-induced transcription of IRF1 was dependent on the PKC-NF-κB signaling pathway, providing a mechanism for proliferation arrest during diapause. Consistently, *in vivo* overexpression of IRF1 in diapause embryos blocked reactivation after restoration of developmental temperatures.

**Conclusions:**

We concluded that embryonic diapause in chicken is characterized by proliferation arrest, which is the same with other spices. However, chicken embryonic diapause is strictly correlated with the cold stress signal and mediated by PKC-NF-κB-IRF1 signaling, which distinguish chicken diapause from the mTOR based diapause in mammals.

**Supplementary Information:**

The online version contains supplementary material available at 10.1186/s12915-023-01550-0.

## Background

Embryonic diapause, a reversible arrest of the development, is a fascinating phenomenon that occurs in the blastocyst stage of embryos [1]. Upon diapause, developing embryos are arrested reversibly for a period and can be reactivated to restore embryonic development without adverse effects [1–3]. This fascinating phenomenon is widely present among unrelated taxa, ranging from plants to insects to vertebrates. Studies have shown that the initiation, maintenance and termination of diapause are regulated by cascades of environmental, hypophyseal, ovarian and uterine mechanisms that vary widely among species [2, 3]. In invertebrates, environmental factors, such as temperature, directly affect the exposed embryo, thereby triggering the diapause state [4]. Alternatively, the regulation of embryonic diapause in viviparous vertebrates requires coordination and crosstalk between the maternal organism and the blastocyst. Nonetheless, environmental signals, such as the temperature and photoperiod, can also influence the diapause state of mammalian embryos [2, 3], thus emphasizing the importance of further examining the effects of environmental stimuli.

To overcome these adverse factors, decreased metabolism and a dramatic reduction or cessation of cell proliferation occur in embryos, resulting in a diapause state. The initiation, maintenance and termination of embryonic diapause involve an intricate succession of genetic and cellular regulatory mechanisms [5–8]. Therefore, uncovering the molecular control of diapause is of particular interest because of its unique growth control and reversibility. Investigation of the mouse blastocyst showed that inhibition of mTOR, a major nutrient sensor and promoter of growth, induces a diapause-like state [9]. Additionally, upstream regulators of mTOR, such as Let-7 [10], c-MYC [11, 12] and Lkb1-AMPK [6], have been demonstrated to regulate mTOR pathway and thus influence mouse embryonic diapause, further highlighting the critical role of mTOR in embryonic diapause in mammals.

The mouse model of diapause appears to be the best studied among the animal models [6, 13]. However, many other species display unique features of embryonic diapause. The fowl retains some characteristics of its reptilian ancestors, especially in terms of the oviparous reproduction strategy. With regard to the chicken, the initial 25 hours of embryonic development occur in the oviduct [14]. The fresh oviposited fertilized egg comprises a 25-hour-old embryo, which is in the blastodermal stage [15]. With regard to the chicken blastoderm, growth arrest (diapause) occurs when the temperature is lower than physiological zero (21°C) [15, 16]. In this state, chicken embryos retain their capability to develop normally after a limited time (< 15 days) of low temperature storage. The embryos recover development when eggs are incubated at a temperature of 37.8°C [8]. The initiation and termination of chicken embryonic diapause is critically determined by the ambient temperature without maternal control. Therefore, chickens are an excellent *in vitro*-like *in vivo* system for extensive investigation of embryonic diapause [8]. Despite these favorable features, the regulators and signaling pathways responsible for chicken embryonic diapause are largely uncharacterized at the cellular and molecular levels.

In this study, we aimed to uncover the molecular control mechanisms for embryonic diapause in the chicken. We used a detailed time course and analyzed pre-diapause, diapause and reactivated embryo states. A comprehensive and integrated gene expression and protein phosphorylation dataset that provides insight into the complex molecular control of embryonic diapause was generated. We identified potential new pathways and genes that regulate embryonic diapause in the chicken.

## Results

### Chicken embryonic diapause is characterized by proliferation arrest

**Fig. 1:**
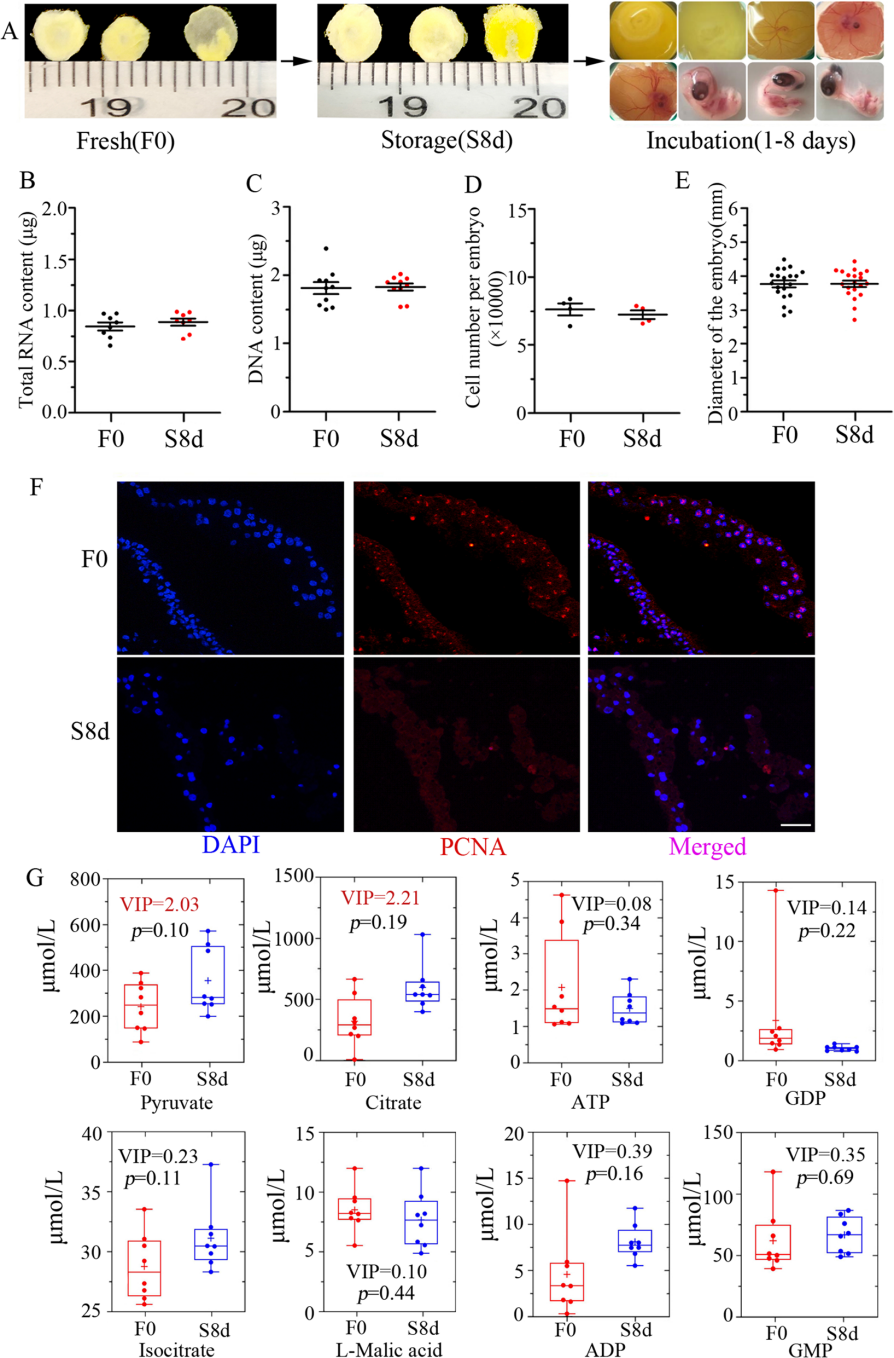
Characteristic differences between pre-diapause and diapause embryos. **A** Morphological examination of the blastoderm at different states. **B**, **C** Amount of extracted RNA (**B**) and DNA (**C**) from each embryo. **D** Total cell number counted by flow cytometry. **E** The diameter of blastoderms. **F** Immunostaining of blastoderm sections with an antibody against the mitosis marker phospho-Histone H3 (H3S10P). Samples were counterstained with DAPI to visualize DNA (blue). Scale bar, 100 μm. **G**, Box plots show the abundance of representative metabolites at F0 or S8d blastoderms. Solid lines in the boxes indicate the median abundance, and the + symbols indicate the mean abundance. The VIP value obtained by OPLS-DA and the *p* value obtained by Student’s t test are provided. See also Additional file 1: Fig. S1C

Fresh oviposited fertilized chicken eggs were able be stored for several days without damage to developmental viability (Fig. 1A). Proliferation arrest and reduced metabolism are distinctive features of the dormant mammalian blastocyst [6]. Therefore, we aimed to evaluate potential differences in cell proliferation and energy metabolism between pre-diapause (fresh oviposited eggs, F0) and diapause chicken embryos (stored at 16°C for 8 days, S8d). First, same number of embryos from pre-diapause and diapause groups were collected, DNA and total RNA were extracted and purified, then quantified using nanodrop 2000. Results showed that the content of RNA and DNA were not changed (Fig. 1B, C). Also, same number of embryos were collected and then digested into single cells and stained with PI, then cell number were evaluated by counting the PI positive cells using FACS, results also showed no significant difference between groups (Fig. 1D). Moreover, the size of the embryos was also evaluated and results showed no significant differences (Fig. 1E), which suggested that the embryos were in a growth arrest state during the storage period. Immunofluorescence staining showed that the mitosis marker phospho-Histone H3 (H3S10P) was undetectable in diapause embryos (Fig. 1F), as well as a drastically reduced PCNA signal (Additional file 1: Fig. S1A), suggested a mitotic arrest in diapause embryos. Additionally, qRT-PCR showed that mRNA expressions of the cell proliferation gene markers MKI67, CCND2 and FOXM1 were significantly suppressed in diapause embryos (Additional file 1: Fig. S1B). Moreover, the TUNEL assay detected a relatively low apoptotic cell number in pre-diapause and diapause embryos (Additional file 1: Fig. S1C). Taken together, these data indicate that chicken diapause embryos undergo cell proliferation arrest.

To determine the metabolic states of pre-diapause and diapause embryos, we profiled the absolute abundance of more than 20 metabolites from the tricarboxylic acid cycle and glycolytic pathway using targeted metabolomics. Orthogonal partial least-squares discriminant analysis (OPLS-DA) identified 4 metabolites with a variable importance projection (VIP) > 1, including pyruvate, citrate, β-D-fructose 6-phosphate and adenosine monophosphate. However, none of them were significantly changed as shown by Student’s *t* test analysis (p > 0.05) (Fig. 1G, and others in Additional file 1: Fig. S1D). Furthermore, the mRNA expression of genes that encode key enzyme in the TCA cycle were not significantly regulated in the diapause blastoderm (Additional file 1: Fig. S1E). The abundance of these metabolites reflected an active metabolism progress in diapause embryos.

### Diapause-altered genes are enriched in proliferation arrest and the stress response

**Fig. 2:**
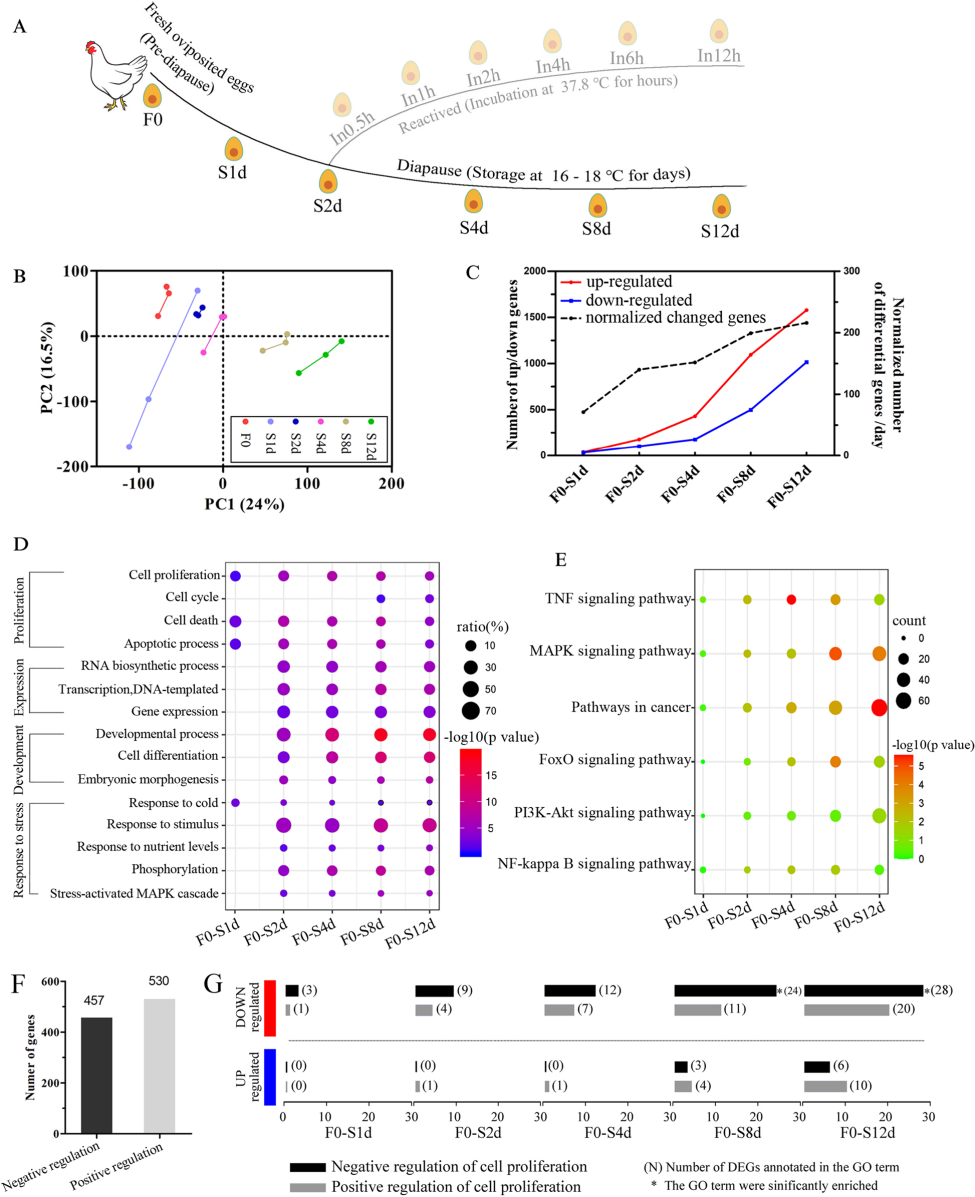
Overview of transcriptomic changes during the onset and maintenance of diapause. **A** Schematic diagram depicting the RNA-Seq workflow time course. Blastoderms isolated from fresh oviposited eggs and low temperature-stored eggs represent pre-diapause and diapause embryos, respectively. Embryos in the diapause state are indicated as S1d, S2d, S4d, S8d and S12d, corresponding to 1, 2, 4, 8 and 12 days of storage, respectively. **B** PCA displays overall gene expression profiles across samples in (**A**). **C** Number of DEGs identified between blastoderms isolated from fresh oviposited eggs and low temperature-stored eggs. DEGs were screened under the threshold of an FDR < 0.01 and |log2fold change| >1. The red and blue lines show the numbers of upregulated and downregulated genes, respectively. The dashed line shows the number of DEGs normalized by the number of interval days. **D**, **E** GO enrichment (**D**) and KEGG pathway (**E**) analysis of DEGs. **F** Number of annotated genes that undergo negative or positive regulation of cell proliferation. **G** A summary of DEGs annotated for negative or positive regulation of cell proliferation

Although no morphological changes occurred within the embryo during the transition between pre-diapause and diapause, we hypothesized that embryonic diapause leads to changes within the transcriptome. Therefore, we used Illumina RNA-sequencing to characterize the transcriptional profiles of embryos in pre-diapause (F0) and diapause (S1d–S12d; Fig. 2A). On average, we obtained 50.6 million clean reads for each sample (Additional file 1: Fig. S2A). Principal component analysis (PCA) showed that the transcriptomes were not randomly disposed, but rather followed a path (Fig. 2B), which is consistent with their temporal order throughout development. Differential expression analysis (with a stringent cutoff of a false discovery rate [FDR] < 0.01 and fold change > 2) showed that 18.5% (3256) of the detected genes (17 613) changed in abundance over the time course, and 16.3% (2 879) changed compared with pre-diapause (F0) embryos (Fig. 2C). Importantly, more than 80% of the identified differentially expressed genes (DEGs) (compared with F0) at each time point were further identified at the next time point (Additional file 1: Fig. S2B), thus further validating the association of identified DEGs with the development of diapause. As additional verification, we examined the dynamics of gene expression across stages to determine whether expression turnover is continuous across different time points. A gradual increase in the number of normalized DEGs suggested that the initiation of diapause followed a pattern of gradual change, rather than a binary switch on/off pattern (Fig. 2C). Together, these data suggest that the gene expression profiles are significantly modulated in chicken embryos during the transition from pre-diapause to diapause.

To gain insight into the biological functions of diapause-altered genes, we performed GO and KEGG enrichment analyses of the DEGs. GO analysis showed known biological processes involved in blastoderm diapause, such as cell proliferation, gene expression and developmental-related processes [17, 18]. The expression profiles of specific genes involved in regulation of proliferation are shown in Additional file 1: Fig. S2C. Furthermore, several novel biological processes were identified, such as MAPK signaling, phosphorylation and the response to stress (Fig. 2D). Notably, the set of GO terms that were significantly enriched (*p* < 0.05) after 1 day of storage were limited to cell proliferation, cell death, the apoptotic process and the response to cold. This finding indicates that genes that function within these processes have the most rapid response to low temperature and may be integral to development arrest. A KEGG pathway enrichment analysis suggested that the DEGs were enriched for the TNF, MAPK, FoxO and NF-kappa B (NF-κB) signaling pathways, among others (Fig. 2E). Remarkably, these signaling pathways are each associated with diverse stress responses [19–21]. The MAPK signaling pathway responds to intracellular and extracellular stresses, including UV light, heat and inflammatory cytokines [21]. NF-κB widely responds to physical, physiological and oxidative stresses, such as heat, viral infection and DNA damage [22]. These data further supported the potential role for stress response genes in diapause.

To better understand the effects of the DEGs on cell proliferation, we examined the upregulated genes and downregulated genes in detail. As shown in Fig. 2F, among the annotated genes in the genome, 457 are involved in negative regulation of proliferation and 530 are involved in positive regulation of proliferation (gene annotations were obtained from the AmiGO 2 database [23]. For the upregulated genes from our analysis, more DEGs are involved in the negative regulation than the positive regulation of cell proliferation, while the pattern was reversed for downregulated genes (Fig. 2G). These results indicate that in the diapause state, genes promoting cell proliferation tend to be repressed while genes preventing cell proliferation tend to be activated, which supports the observed pattern of cell proliferation arrest. The expression profiles of specific genes involved in negative regulation proliferation (marked in red), including IRF1, PPP1R8, TGFB2 and SKI, and genes FOXM1, CCND2, CDKN1A which are involved in negative regulation proliferation (marked in blue) are shown in Additional file 1: Fig. S2C. These results suggested that genes altered by diapause are enriched in proliferation arrest.

We also examined genes associated with pluripotency because maintaining pluripotency of embryonic stem cells is essential for developmental recovery after diapause. As expected, diapause embryos retained an active pluripotency network, highlighted by the expression of the pluripotency markers *POUV (Pou5f3), NANOG, Sox-2* and *Lin-28* (Additional file 1: Fig. S2D). Taken together, these results indicate that low temperature-induced embryonic diapause is characterized by a continuous stream-like progression of cell proliferation arrest without obvious effects on the pluripotency of embryonic stem cells.

### Embryonic diapause is reversed within 2 hours of incubation

**Fig. 3:**
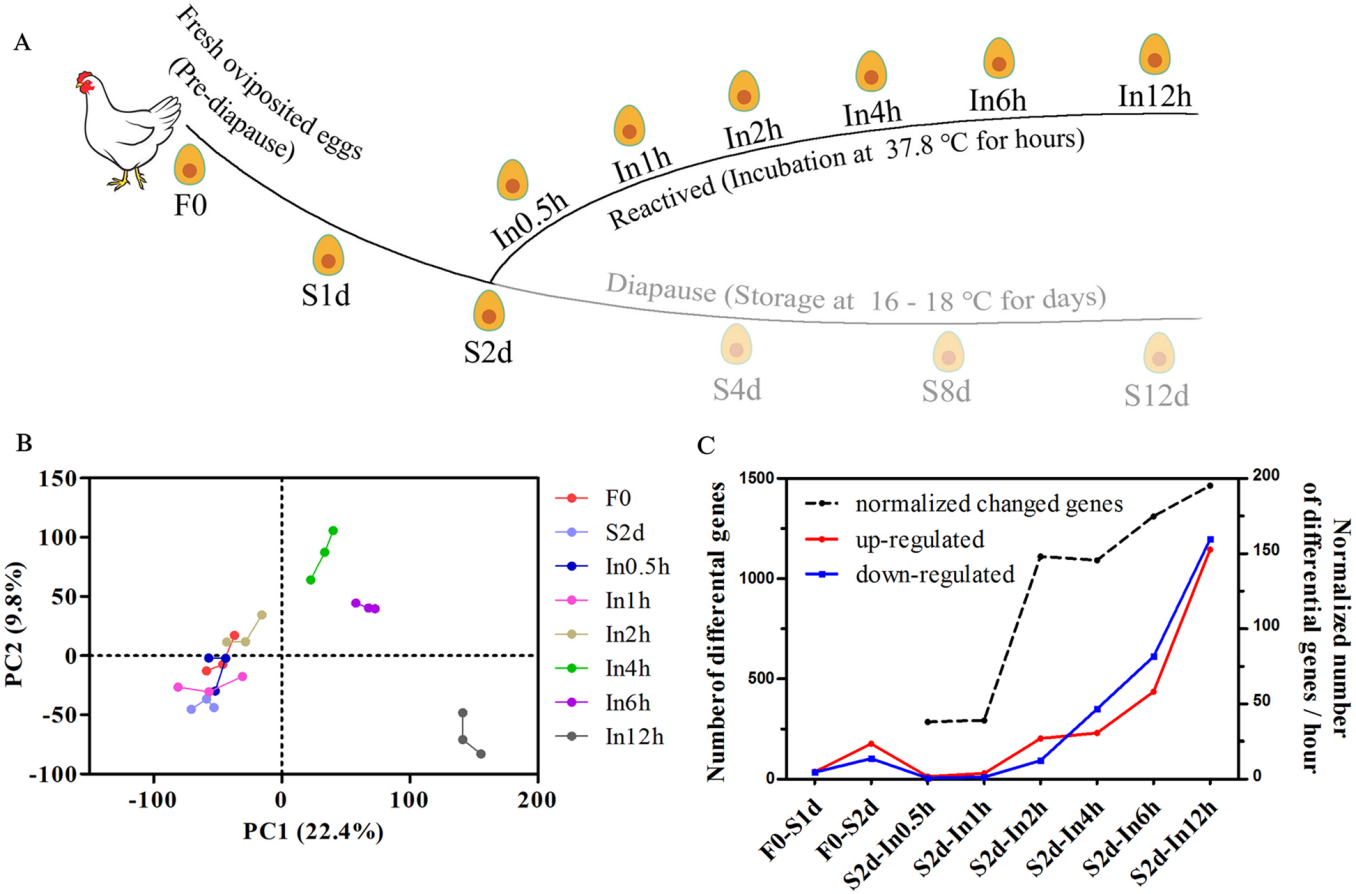
Transcriptomic analysis of the reactivation process after diapause. **A** Schematic diagram depicting the RNA-Seq workflow time course. Eggs were stored at low temperature for 2 days and then incubated at 37.8°C for 0.5–12 hours. The 37.8°C incubation represents the reactivation process. **B** PCA of overall gene expression profiles across samples in panel A. **C** Number of DEGs identified between blastoderms isolated from fresh oviposited eggs, low temperature-stored eggs or incubated eggs. DEGs were screened under the threshold of an FDR < 0.01 and |log2fold change| >1. The red and blue lines show upregulated and downregulated genes, respectively. The dashed line shows the number of DEGs normalized by the number of interval hours

Low temperature-induced embryonic diapause can be reversed when the temperature is returned to approximately 37.8°C. Therefore, we speculated that a global view of the reactivation process would help to identify the molecular controls of the reversible diapause stage. We collected embryos undergoing reactivation and subjected them to RNA-Seq analysis. Importantly, the time point for ending embryonic diapause or beginning reactivation is difficult to define. We previously reported that dramatic transcriptomic changes occur during the first day of incubation [24], but some initial detects can be observed after 6–7 hours of incubation [25]. Because of the short time window to capture the reactivation process of embryonic diapause, we designed a detailed time course to provide an expansive view of the reactivation process (Fig. 3A). Chilled eggs (S2d group, stored at 16°C for 2 days) were transferred into a specialized incubator for 0.5, 1, 2, 4, 6, and 12 hours (In0.5h, In1h, In2h, In4h, In6h and In12h, respectively). A detailed description of the sample quality control parameters is shown in Additional file 1: Fig. S3A. PCA analysis showed that In4h, In6h and In12h embryos were distinctly separated from other groups (Fig. 3B), which suggested that a short time window (< 4 hours) may have preceded the initiation of the reactivation process. Importantly, samples incubated for shorter times (In0.5h–2h) were closer to pre-diapause (F0) embryos than the ln 4–12h embryos, which suggested > 2 hours were required to break diapause and recover development. These results were further confirmed by differential expression analysis. Relatively few DEGs were observed in the initial 1 hour of incubation (In1h) compared with S2d embryos (< 50 genes), but the most DEGs were detected between S2d and In12h embryos (> 2000 genes, Fig. 3C). When the numbers of DEGs were normalized by the intermittent hours, dramatically increased numbers of normalized DEGs were found during the second hour of incubation (Fig. 3C). This finding further verified the time point (In2h–In4h) for diapause embryos to “wake up”. Taken together, our results suggest that In2h–In4h is a crucial time for ending diapause, thus laying the foundation for subsequent developmental progress.

### Diapause-altered genes are conversely regulated during reactivation

**Fig. 4:**
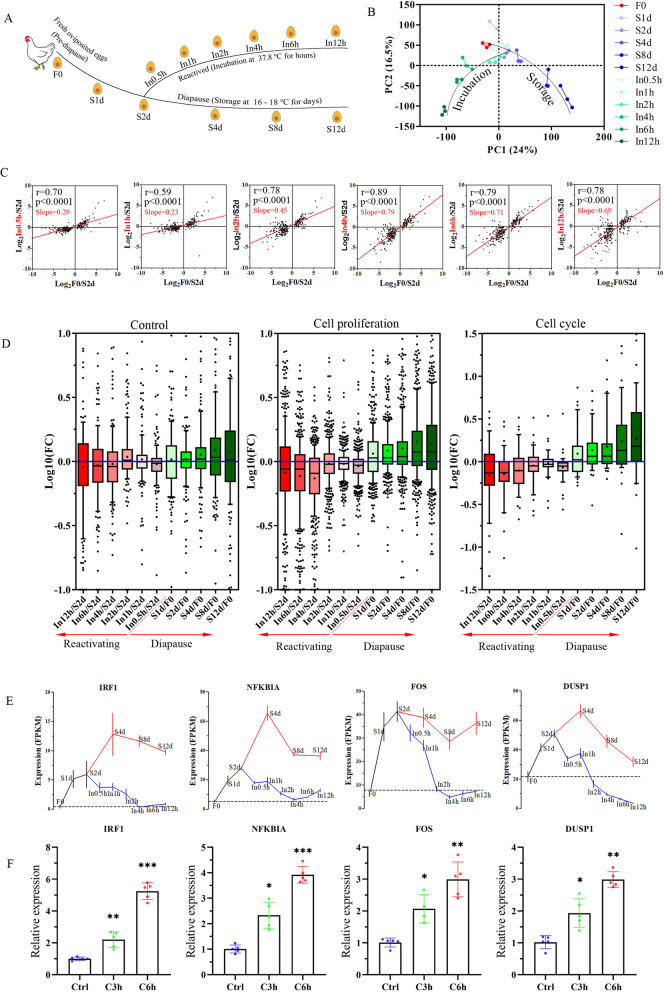
Integrated transcriptomic analysis of the onset, maintenance and termination of diapause. **A** Schematic diagram shows the RNA-Seq workflow time course. **B** PCA displays gene expression profiles across all sequenced samples. **C** Scatterplot for comparing the fold change of gene expression during the reactivation process. Each dot represents a DEG identified by comparing pre-diapause embryos (F0) with diapause embryos (S2d). The x-axis shows the log2fold change in expression in pre-diapause embryos (F0) compared with diapause embryos (S2d). The y-axis shows the log2fold change of expression in reactivating embryos (In0.5h–In12h) compared with diapause embryos (S2d). Linear regression analysis was performed for each panel. The Pearson R, p value and slope of the regression line are indicated. **D** Boxplots show the expression profile of genes that are annotated in cell proliferation and the cell cycle. The y-axis represents the log10 fold change. The horizontal blue line is the zero line of log10 (fold change). Black lines in the boxes indicate the median of log10 (fold change), and the + symbols in the boxes indicate the mean of log10 (fold change). A gene set comprising 150 random selected genes served as a control. **E** RNA-Seq data show dynamic changes in expression of genes at different time points. The dashed line indicates the expression level in F0 embryos, *N* = 3. **F** qPCR of the changes in gene expression after embryonic stem cells were induced by cold stress (28°C) for 3 hours (C3h) or 6 hours (C6h), *N* = 5. *p < 0.05; **p < 0.01; ***p < 0.001. N indicates biological replicates

To provide an additional important metric for transcriptomic divergence, we visualized PCA results using all sequenced samples (Fig. 4A, B). Generally, the F0, S1d-S2d and In0.5h–In2h samples were slightly separated from each other. However, the reactivated embryos (In4h–In12h) and long-term diapause embryos (S4d–S12d) followed a distinct path, which is similar to the experimental design shown in Fig. 4A. Therefore, we hypothesized that diapause-altered genes are conversely regulated during the reactivation process. To affirm this possibility, we examined a set of 280 DEGs which is identified from comparation between pre-diapause and diapause states (S2d vs F0). The log2fold changes in pre-diapause/diapause and reactivation/diapause for these gene were evaluated by linear regression analysis. All scatter diagrams generated during the reactivation process showed a positive correlation (Pearson R > 0.5, *p* value < 0.0001; Fig. 4C). Interestingly, the regression line had a relatively low slope (slope < 0.3) in the first hour of incubation, followed by an obviously increased slope (slope = 0.45) after 2 hours of incubation. The slope was highest (slope = 0.79) after 4 hours of incubation, which suggested that the differences in these genes progressively increased and were greatest after 4 hours. Consistent similar results were observed when all expressed genes (average FPKM > 0.1) were analyzed (Additional file 1: Fig. S3B). To obtain additional mechanistic insight, we examined the expression profiles of genes annotated in specific GO terms. As expected, the significantly enriched GO terms of cell proliferation and the cell cycle were conversely regulated during the reactivation process (Fig. 4D). These results indicate that the expression profile during reactivation is essentially reversed.

### *IRF1, FOS, DUSP1* and *NFKBIA* are markers of chicken embryonic diapause

As an additional goal of this study, we aimed to identify regulators that may be responsible for embryonic diapause. Because of the continuous stream-like progression between pre-diapause and diapause, we considered that these regulators should rapidly respond to a change in temperature and display a converted expression pattern with the transition of embryonic states as shown in Fig. 4A. We have identified 33 known genes that are differentially expressed between F0 and S1d groups (4 genes shown in Fig. 4E and 29 genes in Additional file 1: Fig. S4A). We then closely examined these genes and filtered these genes against the following conditions: (i) the expression level in diapause embryos (S1d–S12d) was always higher (lower) than that in F0; (ii), the expression level in incubated embryos was not higher (lower) than that in S2d; and (iii) the expression level in In4h was similar to that in F0.

As a result, 19 candidate genes were identified (4 genes in Fig. 4E and the first 15 genes in Additional file 1: Fig. S4A). The 19 genes are significantly implicated in cell proliferation and stimuli response (Additional file 1: Fig. S4B), which is consistent with our previous findings. Among them, five genes (*IRF1, NFKBIA, SOX7, NME1* and *ADM*) are involved in negative regulation of cell proliferation, and five genes (*GDNF, NOV* [also known as *CTGF* or *CCN2*], *SOX8, FOS* and *GADD45G*) are involved in negative regulation of cell death. Four of these genes (*IRF1, GADD45G, JUN* and *DUSP1*) are involved in regulation of the cell cycle, and nine genes (*IRF1, FOS, NFKBIA, NME1, ADM, NOV, GADD45G, JUN* and *DUSP1*) are involved in the stress/stimulus response. Additionally, two genes (*FOS* and *NFKBIA*) are involved in response to cold stress. Interaction enrichment showed that 10 of the 19 genes significantly interacted with each other (*p* < 0.001, Additional file 1: Fig. S4C), which indicated that these genes are functionally connected. To further identify cold stress responsive genes in cells, isolated chicken embryonic stem cells (cESCs) were exposed at a low temperature of 28°C for 3 and 6 hours. A qPCR analysis showed that 4 of the 19 genes, namely *IRF1, NFKBIA, FOS* and *DUSP1*, were significantly upregulated by the cold stress (Fig. 4F and Additional file 1: Fig. S4D). Collectively, elevated transcription of these genes may serve as a temperature-sensitive marker of embryonic diapause in the chicken embryo.

### Phosphoproteomics indicates the implications of PKC in diapause

**Fig. 5:**
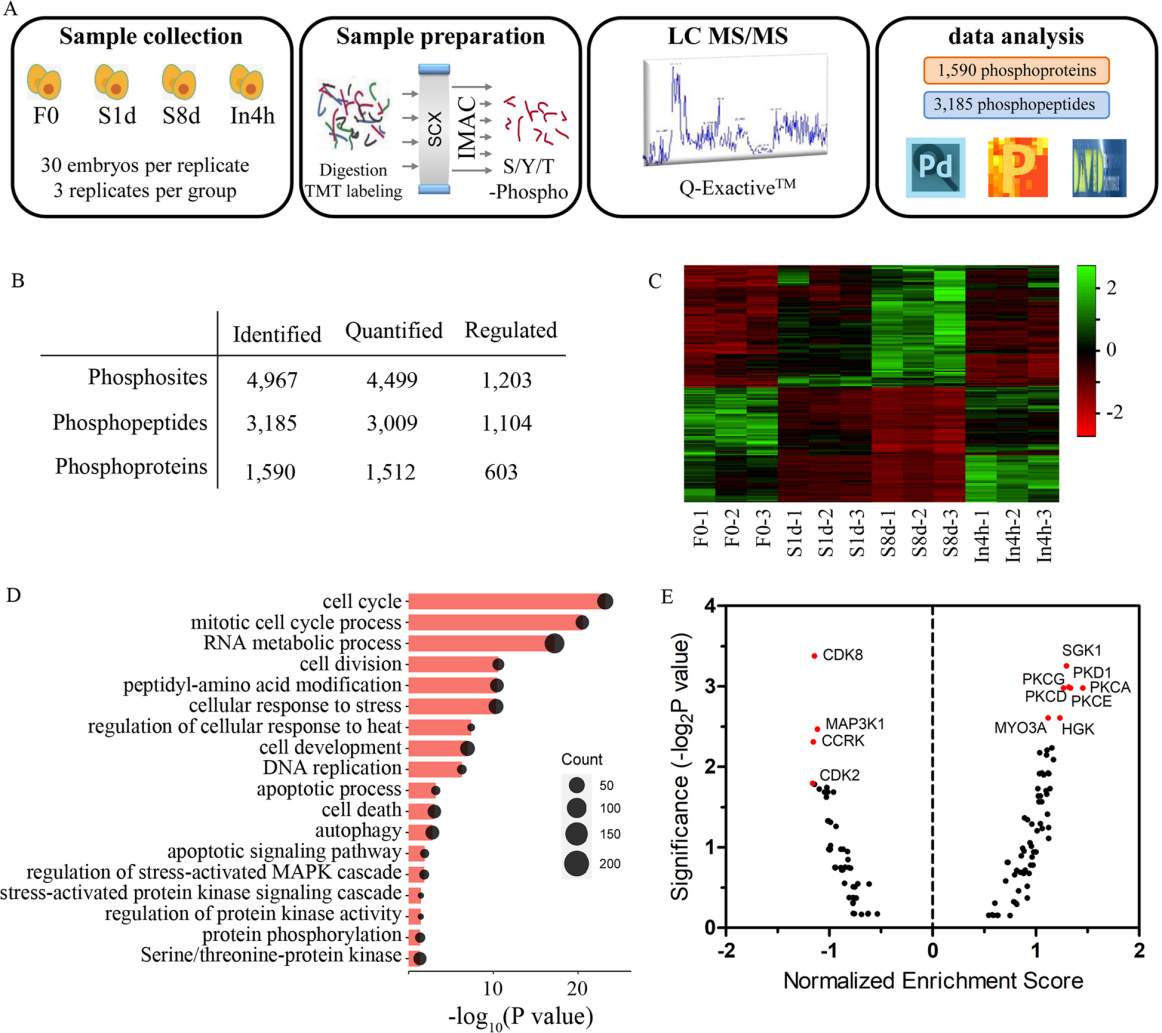
Phosphoproteomic analysis of low temperature-induced diapause. **A** Schematic diagram depicting the phosphoproteome workflow. **B** Data summary of the identified phosphoproteins and peptides. **C** Heatmap of phosphorylation levels of significantly regulated phosphopeptides among the groups. **D** DAVID analysis of enriched GO terms for significantly regulated phosphoproteins. **E** Enrichment score and significance of all protein kinases obtained by kinase-substrate enrichment analysis on the basis of diapause-regulated phosphopeptides. A normalized enrichment score > 1 indicates a positive enrichment in the diapause group, while a normalized enrichment score < 1 indicates a negative enrichment in the diapause group

The rapid transcriptional induction of IRF1, NFKBIA, FOS and DUSP1 is presumably regulated by post-translational modification of proteins. Therefore, we focused on prompt and directly regulated protein phosphorylation, which is shown to be implicated in diapause (Fig. 2D). To understand the phosphorylation status of proteins in embryos under diapause, phosphoproteomic analysis of the F0, S1d, S8d and In4h groups was performed (Fig. 5A). Using 12 LC-MS/MS measurements, we quantified more than 3000 phosphopeptides from 1512 proteins (Fig. 5B). The quantified phosphoproteome was highly reproducible with an average Pearson’s correlation coefficient between all measured samples of 0.89 (Additional file 1: Fig. S5A). A PCA analysis clearly classified the different groups (Additional file 1: Fig. S5B). In agreement with other MS-based studies [26], the majority of phosphorylation events occurred on serine residues (86%), followed by threonine (12.6%), whereas phosphotyrosines accounted for 1.4% of quantified phosphosites (Additional file 1: Fig. S5C). Differential analysis showed that 34.7% of quantified phosphopeptides significantly increased or decreased in abundance in at least one comparison (Additional file 1: Fig. S5D). Similar to the gene expression profile, the diapause-regulated phosphopeptides were reversely regulated after incubation (Fig. 5C). Functional enrichment showed that regulated phosphoproteins were significantly enriched for the cell cycle and response to stress-associated categories (Fig. 5D), which were also highlighted by our transcriptomic data. Specifically, the phosphorylation level of major proteins involved in the cell cycle, such as NDRG1 RANBP1, GAB1, BCL9, were significantly regulated (Additional file 1: Fig. S5E).

To investigate the protein kinase responsible for changes in the phosphoproteome, we performed kinase enrichment on the basis of regulated phosphopeptides. The increased phosphopeptides in diapause embryos were enriched in kinase motifs for PKC, specifically the typical family members PKCα and PKCε (Fig. 5E). This finding is interesting because PKC kinases have an explicit role in the stress response, cell proliferation and cell survival [27], and PKC is thought to be activated by cold stress [28]. Additionally, an increased PKC activity under diapause was supported by the phosphoproteome, which showed that phosphorylation levels of the well-known PKC substrates Marcks and MarcksL1 were significantly increased in the diapause state (Additional file 1: Fig. S5F).

Previous studies have reported that the mTOR signaling pathway is a critical element in inducing mouse blastocyst diapause [9], and we therefore examined the potential role for this pathway in chicken diapause. First, we examined our transcriptomic data, results showed that most of the genes in the mTOR signaling pathway (collected from PID Pathways [29]) were not changed, including the mTOR activator AKT1 and inhibitor TSC2 (Additional file 1: Fig. S6A). Then we examined our phosphorylation quantitative data, a total of 42 phosphorylation sites in mTOR target genes were quantified. Only 6 sites showed significant difference between F0 and S7d, but none of these sites showed significant difference between F0 and S1d (Additional file 1: Fig. S6B). Further, western blot showed that phosphorylated form of ribosome protein S6 (pS6), which is a reliable marker for mTOR activation, is not detected both in F0 and S1d (Additional file 1: Fig. S6C). These results suggested that mTOR is not responsible for the pre-diapause and diapause transition.

### Upregulation of our four potential marker genes is dependent on PKC-NF-κB signaling

**Fig. 6:**
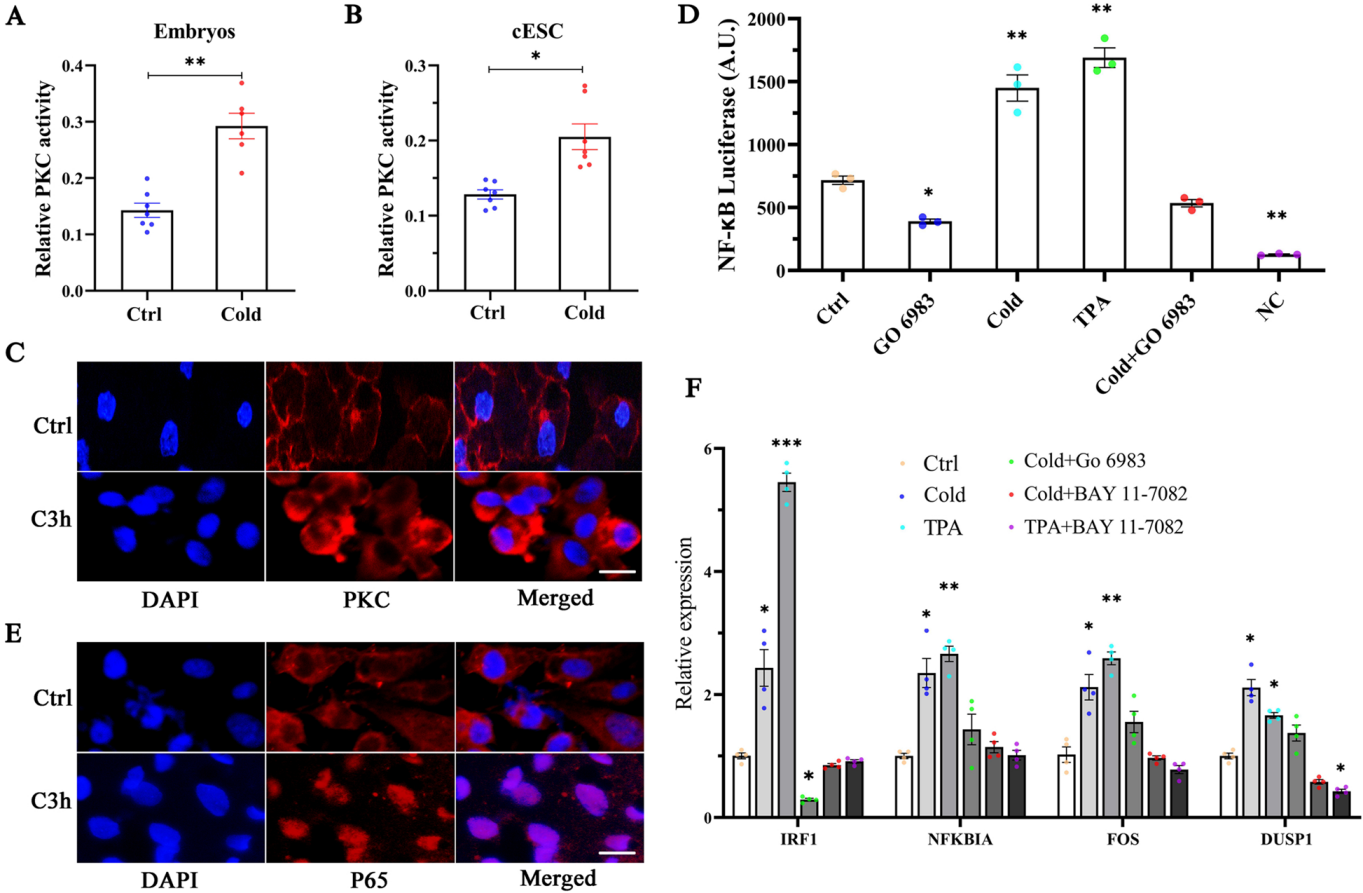
Rapid induction of IRF1 transcription is mediated by PKC-NF-kB signaling. **A** PKC activity levels in chicken embryos treated with cold stimulation (28°C) for 12 hours, *N* = 7. **B** PKC activity levels in cESCs treated with cold stimulation (28°C) for 3 hours, *N* = 7. **C** Immunostaining of cESCs with or without 3 hours of cold stimuli. Staining was performed with an antibody against PKC (green). DAPI was applied as a counterstain (blue). Scale: 20 μm. **D** NF-kB luciferase levels in cESCs treated with cold stimulation (28°C), a PKC activator (TPA) or a PKC inhibitor (GO 6983) for 3 hours. cESCs transfected with an NF-kB luciferase reporter vector and untransfected cESCs served as the control group (Ctrl) and negative control group (NC), respectively, *N* = 3. **E** Immunostaining of cESCs with or without 3 hours of cold stimuli. Staining was performed with an antibody against P65 (green) or DAPI (blue). Scale: 20 μm. **F** Changes in gene expression after treatment with cold stimuli, and an activator and/or inhibitor of PKC and NF-kB for 3 hours in cESCs, *N* = 4. The mean ± SEM is shown in all panels. *p < 0.05; **p < 0.01; ***p < 0.001. N indicates biological replicates

PKC is a well-known protein kinase directly involved in NF-κB activation [30–33]. Importantly, all of our four genes are known NF-κB target genes (TD Gilmore Database), and NFKBIA is a prototypical direct target for an early response of NF-κB [34]. Therefore, we hypothesized that low temperature-induced *IRF1*, *NFKBIA*, *FOS* and *DUSP1* transcription is dependent on PKC activity. Consistent with this possibility, cold stimulation increased PKC activity in chicken embryos and cESCs (Fig. 6A, B), and increased plasmalemma localization of PKC (Fig. 6C). Additionally, cold stimulation increased the transcriptional activity of NF-κB, the nuclear translocation of p65, and the transcription of IRF1, NFKBIA, FOS and DUSP1 in cESCs (Fig. 6D - F), as well as several other known NF-κB targeted genes (*TNFAIP3, NFKBIZ, IL1B, IL8* and *CD83*; Additional file 1: Fig. S7). As expected, the potent PKC activator 12-O-tetradecanoylphorbol13-acetate (TPA) had a similar effect as cold in increasing NF-κB transcriptional activity. However, GO 6983 (pan-PKC inhibitor) and BAY 11-7082 (inhibitor of the NF-κB pathway), significantly blunted the cold stress-induced NF-κB transcriptional activity (Fig. 6D, F). These findings confirmed that the cold-induced transcription of these genes is dependent on PKC activity. These data suggest that PKC-NF-κB signaling is integrally involved in chicken embryonic diapause.

### IRF1 is a negative regulator of cell proliferation

**Fig. 7:**
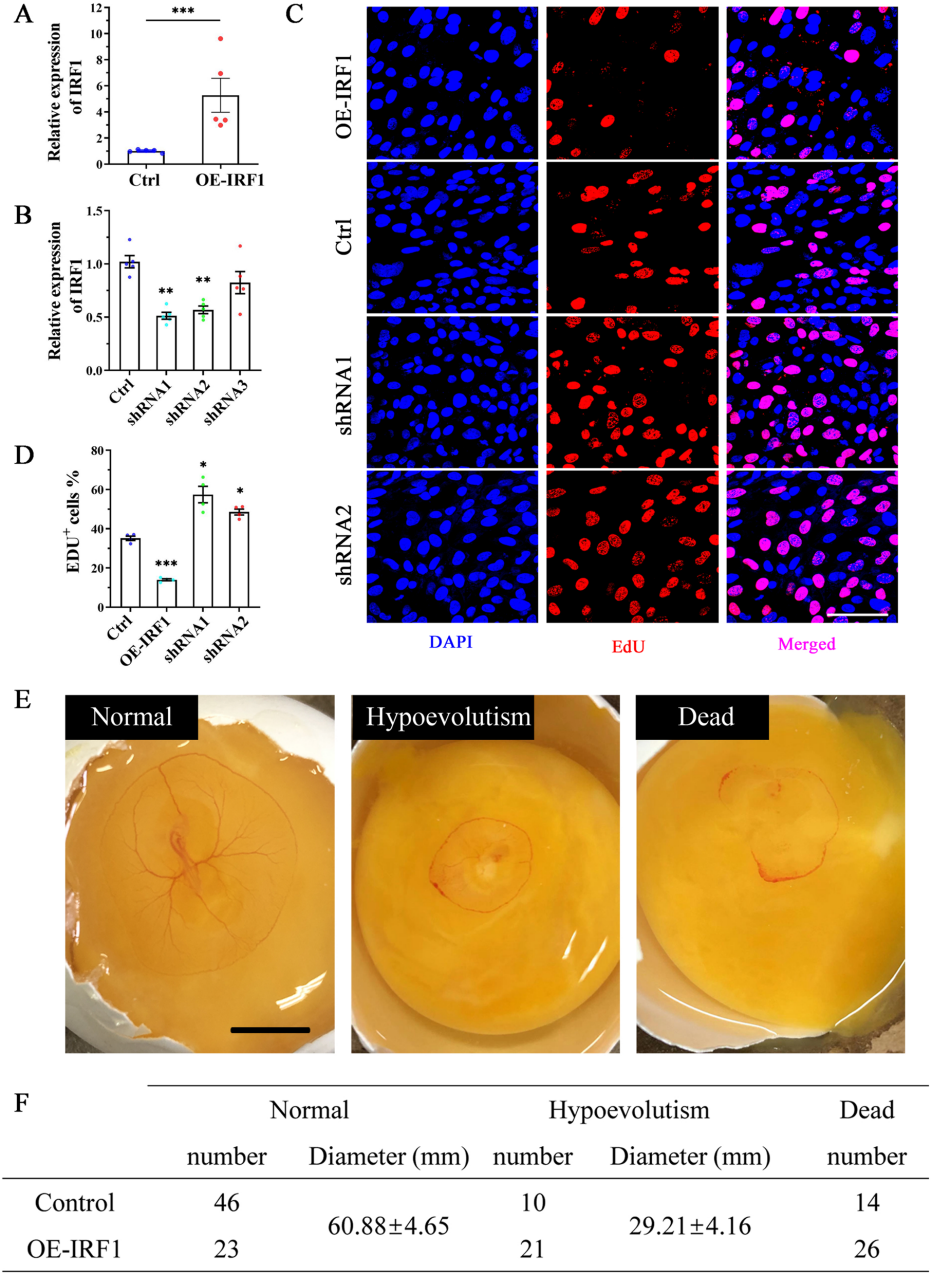
IRF1 inhibits cell proliferation and embryonic growth. **A**, **B** Efficacy of lentiviral-mediated IRF1 overexpression (OE-IRF1) (**A**) or knockdown (shRNA) (**B**) in DF1 cells, N = 5. **C** Representative confocal microscope images of EdU incorporation. Scale: 20 μm. **D** Quantification of panel C. Values are shown as the mean ± SEM, N = 4. **E** Representative images of embryos generated from incubated eggs on day 3. The eggs were stored at 16°C, and injected with the OE-IRF1 lentivirus to overexpress IRF1 or injected with the negative control lentivirus. Two days later, injected eggs were transferred to an incubator and incubated at 37.8°C for 3 days. Scale: 1 cm. **F** Statistics of different phenotypes shown in panel E

Among the 4 genes downstream of PKC-NF-κB signaling pathway, NFKBIA function as negative feedback regulators to control NF-κB signaling dynamics [35]. DUSP1 was reported to have a role in maintains IRF1 expression and therefor leads to control the expression of IRF1-dependent genes [36]. Therefore, we focused on the most downstream gene IRF1, which is known as a negative regulator of cell proliferation [37] and most increased after cold stimulation (Fig. 4F). To determine the specific role of IRF1 in cell proliferation, we overexpressed and knocked down IRF1 in the cell line DF1. DF1 was used instead of cESCs for these experiments because cESCs proliferate slowly *in vitro* and are unsuitable for long-term culture. As expected, infection of DF1 cells with a recombinant overexpression lentivirus was effective in driving IRF1 expression (OE-IRF1), whereas infection with a knockdown lentivirus (shRNA1-3) had the opposite effect (Fig. 7A, B). Furthermore, 5-Ethynyl-2′-deoxyuridine (EdU) proliferation assays showed that cell proliferation was negatively regulated by IRF1 (Fig. 7C, D). These results were verified by FACS analysis of the cell cycle, which suggested that overexpression of IRF1 arrested the cell cycle at the S phase (Additional file 1: Fig. S8A, B). These findings are consistent with an established role of IRF1 in cell cycle regulation [38, 39] and support its potential role in regulating cell proliferation during diapause.

To provide further *in vivo* evidence of the role of IRF1 in cell proliferation in the chicken embryo after recovery from diapause, we investigated whether overexpression of IRF1 delays embryonic development. A recombinant IRF1 overexpression lentivirus and a negative control lentivirus were injected into fertilized eggs. After 48 hours of low temperature storage (16°C), the eggs were sampled and examined. We found that IRF1 mRNA levels were > 10-fold higher in OE-IRF1 embryos than in control embryos (Additional file 1: Fig. S8C), but there was no difference in the size of the blastoderms between the two groups (Additional file 1: Fig. S8D). Subsequently, the remaining postoperative eggs were incubated at 37.8°C, and the embryos were harvested and examined after 3 days. Most (65.7%) of the control embryos were morphologically viable, whereas most of the IRF1 overexpression embryos were smaller (30%) or dead (37.1%) (Fig. 7E, F). Collectively, these results indicate that overexpressed IRF1 delays embryonic development after diapause.

## Discussion

Uncover potential molecular mechanisms, but little is known about the embryonic diapause in avian species. As a valuable vertebrate model system for embryological studies [40, 41]. Chickens retain an oviparous reproduction strategy, with embryonic diapause closely determined by the environmental temperature, thus making chicken embryos an excellent artificially controlled *in vivo*-like *in vitro* model. In this study, we dissected dynamic gene expression with precise time resolution across the transition of embryonic states. Importantly, using transcriptome analysis, we found that diapause embryos gradually recovered development within 4 hours of incubation, and In2h–In4h was identified as a crucial period for diapause termination. These findings lay the foundation for subsequent development of chicken embryos as a novel model for investigating diapause. The ability to artificially control the temperature and precisely control the timing profile of the transition between diapause and development makes chicken embryonic diapause an ideal model for uncovering the mechanism of diapause.

A defining characteristic of diapause in mammalian species is the dramatic reduction or cessation in proliferation of the embryo [2]. In agreement with data from other species, our data showed an abrupt arrest of embryonic cell proliferation. Although cell proliferation arrest plays a causal role in embryonic diapause, the mechanisms for induction of mitotic arrest depend on the species and are poorly understood. Recent studies have shown that proliferation arrest in mouse embryo diapause is related to the inhibition of mTOR signaling [9] or MYC [11]. Other studies have suggested that miRNA Let-7 overexpression in mouse embryos prevents implantation and induces diapause [10, 42]. Interestingly, Let-7 is upstream of mTOR, while MYC is a downstream target of mTOR [10, 43], further indicating the critical role of mTOR signaling in mouse embryonic diapause. In this study, we analyzed the mTOR pathway activity by integrating transcriptomic, phosphoproteome data, as well as the WB and immunofluorescence staining of mTOR target pS6. Results suggested that mTOR is not responsible for the pre-diapause and diapause transition. Notably, we observed relatively low expression of pS6 both in pre-diapause and diapause embryos (Fig. S6C), which indicated low mTOR activity in these embryos. The mTOR pathway is the major nutrient-sensitive regulator of cell growth in animals [44]. However, our results showed that the cell proliferation, not cell growth, is the key to control diapause in chicken, which may explain the irrelevance of mTOR in the context of chicken diapause. Moreover, it is interesting that high mTOR activities were observed in longtime stored embryos (Fig. S6C). It’s reported that inhibiting the metabolism and growth of chicken embryos, such as by lowering environmental temperature, can prolong the storage time of chicken eggs [45]. The increased mTOR activity in longtime stored embryos may lead to increased metabolism and growth, and therefore result in unsustainable diapause state. Thus, we conclude that mTOR is not responsible for the induction of diapause but may serve as a critical regulator for maintaining the diapause state in chicken. These results suggest that the mechanisms for chicken embryonic diapause is completely differ from those of the mouse model.

Another well-defined characteristic of diapause in mammalian species is the dramatic decrease in metabolism. Early investigations of diapause in rodent embryos showed a depressed metabolic profile characterized by changes in the glycolytic and pyruvate pathways [6]. Another study also showed the transcriptome profile of diapause that is associated with downregulation of metabolism [18]. Mammalian embryos are highly metabolically and transcriptionally active at the blastocyst stage, while in diapause state, the dramatic decrease in metabolism may be purposed to maintain cell viability and thus ensure the embryo’s survival. Low temperature, on the other hand, may reduce the metabolic enzyme activity and thus the level of metabolism in chicken embryos. However, a low metabolic level is obviously not conducive to cell survival under continuous cold stress. We have detected comparable RNA content in both pre-diapause and diapause embryos; accordingly, no large number of depressed genes were detected in diapause embryos, suggesting an unchanged transcriptional activity during the induction of diapause. Moreover, our results showed a comparable abundance of the TCA metabolites in diapause embryos compared with the pre-diapause embryos. Even though further studies need to better investigate the specific metabolic rate to uncover the role of metabolism in chicken diapause, our data have already shown some differences between chicken and mammalian diapause.

mTOR is one of the most important integrators of nutrient-sensing signals in mammalian cells [46]. Therefore, a lack of mTOR pathway activation in avian versus mammalian embryos may explain the lack of effect on energy metabolism in chicken diapause as demonstrated in this study. The oviparous reproduction strategy of chickens and other avian species is distinct from that of mammalian species in that the development of chicken embryos is fundamentally dissociated from maternal control. Moreover, the embryo is surrounded by the vitellus, which comprises abundant fatty acids, protein, vitamins and mineral substance, thus causing the embryos to be less prone to metabolic stress. These features may distinguish the metabolism regulatory mechanisms of chicken embryonic diapause from those of mammals. Given that metabolites abundance is insufficient to represents the metabolism activity, research on the effects of diapause on metabolism, and its regulatory mechanisms still requires extensive work.

Using unique features of diapause in chickens, we investigated the dynamic transcriptome, including the initiation, maintenance and reactivation of diapause. Our functional enrichment assays identified cell survival-associated signaling pathways, such as the NF-κB and MAPK pathways, and host defense pathways, thus providing new clues to uncover mechanisms for diapause-induced proliferation arrest in oviparous animals. Such transcriptomic information facilitated further extraction of potential candidate genes of which expression characteristics were highly synchronized with embryonic developmental states. Importantly, many of these genes serve as regulators of cell proliferation, cell death and the cell cycle. An example of this regulation is that ectopic overexpression of IRF1 strongly inhibits cell proliferation in several cell types [37, 38]. Consistent with these reports, we found that IRF1 was a master negative regulator of cell proliferation *in vitro*. Fos and Jun, the most potent AP-1 transcription factor subunits, are induced by a broad range of stimuli and regulate cell proliferation, death and survival [47, 48]. Previous studies showed the same result of upregulation of Jun during avian embryonic diapause [8, 49], which suggested that it is a potential regulator of diapause in avian species. Another important gene that may be responsible for the crosstalk between low temperature and diapause is GADD45G, which is a common stress sensor implicated in growth arrest and apoptosis [50]. Identification of these stress responsive and proliferation-associated genes further confirmed the proliferation arrest observed during diapause in the chicken. However, understanding the synergistic effect on proliferation arrest among these genes, and the synergistic effect between proliferation arrest and apoptosis requires additional rigorous investigation. Nonetheless, these results suggest that the growth arrest and survival mediated by stress responsive genes represent critical mechanisms for low temperature-triggered diapause in the chicken embryo.

Because temperature acts as a specific driver of the transition between diapause and development, we screened cold stress-induced genes in cESCs, including *IRF1, NFKBIA, FOS* and *DUSP1*. Notably, IRF1 and FOS are well-known transcriptional regulators of host defense [38, 48]. We found that the expression of these genes was regulated by NF-κB. The transcriptional activation ability of NF-κB is inhibited by heat stress via inhibition of NF-κB nuclear translocation [51]. NF-κB plays a central role in the control of inflammation, proliferation and apoptosis, thereby providing a cell survival mechanism to withstand adverse stress [19, 52]. We found that NF-κB transcriptional activity was induced by cold stress, suggesting that NF-κB is an important regulator of cold stimuli. Moreover, a recent study showed that inflammatory stimuli induced stem cell quiescence via NF-κB [53]. The accumulation of saturated fatty acids in diapause embryos also stimulates NF-κB, and thereby facilitates survival of the diapause embryo [6]. In our study, transcriptomic analyses showed significant enrichment of the NF-κB signaling pathway in diapause embryos, and NF-κB had the upstream regulators *IRF1, NFKBIA, FOS* and *DUSP1*. These findings further confirmed the potential role of NF-κB in embryonic diapause. Future studies on distinct targets of cold-activated NF-κB in cESCs may help pinpoint the exact mechanisms of NF-κB in diapause.

Notably, the induction of these genes by temperature occurs rapidly, and may not require *de novo* protein synthesis. Indeed, the functional diversity of NF-κB and other transcription factors that act as receptors for executing responses to extracellular stimuli is often dependent on modification (e.g., phosphorylation) [38, 54]. Previous studies have shown that specific protein kinase-mediated phosphorylation is a key regulatory mechanism during diapause [5, 8, 55]. In this study, we describe the phosphoproteomic profiles of chicken embryos during transition from diapause to development, and clearly showed that phosphorylation was involved in the cell cycle-related process. Additionally, kinase enrichment showed significant activation of PKC in diapause embryos. PKC is a well-known kinase implicated in direct NF-κB activation [19, 32, 33], and cold stress facilitates inflammatory responses though PKC-NF-κB signaling in primary airway epithelial cells [56]. Consistently, we demonstrated that cold stress in the chicken embryo resulted in proliferation arrest through PKC-NF-κB signaling. These findings suggest that the PKC–NF-κB signal transduction pathway may represent a universal mechanism for cold stimuli in avian, with the responses are likely dependent on cell-specific and species-specific NF-κB targets.

## Conclusions

This study uncovered a PKC-NF-κB-IRF1 signaling based regulation mechanism for chicken embryonic diapause. It is important to note that this mechanism is significantly different from the mTOR signal-based regulatory mechanism recently uncovered in mouse model. Our findings will provide valuable new avenues for exploring the perplexing nature of embryonic growth arrest.

## Methods

### Chicken embryo collection

A pure line of White Leghorns, aged 35–45 weeks, were artificially inseminated. Fertile fresh laid eggs were collected daily and designated as F0. The fresh oviposited eggs were immediately (within 5 minutes) dedicated to blastoderm isolation. Cracked, soiled, poorly shaped, unusually large or small eggs were removed. To induce embryonic diapause, the eggs were stored in a dedicated egg storage room with an automatic temperature control system (16°C and 90% relative humidity) for days (S1d, S2d, S4d, S8d and S12d, corresponding to 1, 2, 4, 8 and 12 days of storage, respectively). To terminate embryonic diapause, the stored eggs were transferred to a specialized incubator without pre-heating for 0.5, 1, 2, 4, 6 or 12 hours ( In0.5h, In1h, In2h, In4h, In6h or In12h, respectively). Collected eggs were disinfected with 75% ethanol and then broken to isolate the blastoderm. Isolated blastoderms were dissected in PBS containing 4% DEPC and washed twice to remove the excess yolk. Unbroken blastoderms were flash-frozen and stored at −80°C until later use.

### RNA isolation, reverse transcription and qRT-PCR

To minimize individual variation, three embryos from the same group were randomly pooled together. Total RNA was extracted using TRIZOL reagent, treated with DNase I at 37°C for 10 minutes. The integrity of total RNA was confirmed by gel electrophoresis, and the RNA concentration was determined using a NanoDrop 2000 spectrophotometer (Thermo Fisher Scientific, Wilmington, DE, USA). Qualified RNA samples were reverse-transcribed into cDNA using the PrimeScript™ RT Reagent Kit with gDNA Eraser (Takara, Kyoto, Japan) according to the manufacturer’s protocol. Gene expression levels were detected by qRT-PCR using the SYBR Green (Takara) method in a Lightcycle®96 instrument (Roche, Indianapolis, IN, USA). All qRT-PCR reactions were performed in triplicate, and the chicken β-actin gene was used as a housekeeping gene. Primers were designed using Oligo 6.0 software. The specificity of each primer pair was verified by NCBI Primers-BLAST online programs. Expression levels were measured in terms of the cycle threshold (Ct) and were normalized to the expression of β-actin using the 2^-ΔΔCt^ method.

### Sample preparation for phosphoproteomic analysis

To perform phosphoproteomics, 30 embryos from the same group were randomly pooled together as one sample. The samples were lysed in SDT lysis buffer containing 4% (w/v) SDS, 100 mM Tris -HCl (pH 7.6) and 0.1M DTT. The samples were then centrifuged for 15 minutes at 16,000 × g at 4°C, and the protein concentration was determined by the BCA Protein Assay Kit (Bio-Rad, Hercules, CA, USA). A total of 200 mg of protein was digested using a filter-aided proteome preparation assay [57]. Digested samples were desalted by running through the C18 cartridge (Empore™ SPE Cartridges C18, bed I.D.: 7 mm, volume: 3 ml; Sigma, St. Louis, MI, USA) and dried by a SpeedVac (Thermo Fisher Scientific). A total of 100 μg of peptides in each sample were labeled using TMT reagents (Thermo Fisher Scientific) according to the manufacturer’s instructions. Labeled peptides were fractionated by strong cation exchange (SCX) chromatography using the AKTA purifier system (GE Healthcare, Piscataway, NJ, USA). The enrichment of phosphopeptides was carried out using the sequential IMAC method by the High-SelectTM Fe-NTA Phosphopeptide Enrichment Kit (Thermo Fisher Scientific). After lyophilization, the phosphopeptides were resuspended in 20 μL of loading buffer (0.1% formic acid).

### RNA-Seq library preparation, sequencing and data analysis

RNA-Seq libraries were generated using the NEBNext Ultra^TM^ RNA Library Prep Kit (NEB, Ipswich, MA, USA) according to manufacturer’s recommendations. Libraries were sequenced on the Illumina HiSeq 2500 in 150-bp paired-end mode. Raw data were cleaned using BBmap (v37.90), and quality control was performed using the FastQC package. The clean data were then aligned to the GRCg6a reference genome using Hisat2 (v2.06). Reads with a perfect match or one mismatch were further analyzed and annotated on the basis of the reference genome. StringTie (v2.13) coupled with Ballgown software was used to quantify the FPKM values for each gene. DESeq was used to screen DEGs under a threshold (FDR) < 0.01 and |log2fold change| >1.

### Phosphoproteomic data acquisition and processing

Phosphopeptides were separated by liquid chromatography on the EASY-nLC 1200 system (Thermo Fisher Scientific) for 120 minutes. Briefly, phosphopeptides were loaded onto a reverse phase trap column (Thermo Scientific Acclaim PepMap100; 100 μm × 2 cm, nanoViper C18) connected to a C18-reversed phase analytical column (Thermo Scientific Easy Column; 10-cm long, 75-μm inner diameter, 3 μm resin). The phosphopeptides were separated by reversed-phase chromatography using a binary buffer system consisting of 0.1% formic acid (buffer A) and 80% acetonitrile in 0.1% formic acid (buffer B) at a flow rate of 300 nl/minute. MS data were analyzed on a Q-Exactive HF mass spectrometer (Thermo Fisher Scientific) using a data-dependent top 10 method, with a maximum injection time of 10 ms, scan range of 300–1800 m/z and automatic gain control target of 3e6. Survey scans were acquired at a resolution of 70 000, and the resolution for HCD spectra was set to 17 500. Normalized collision energy was 30 eV, and the underfill ratio was set to 0.1%.

MS data were processed with Proteome Discoverer version 2.4. Enzyme specificity was set to that of trypsin, allowing for cleavage up to two missed cleavage sites. Carbamidomethyl (C), TMT 6/10 plex (N-term) and TMT 6/10/16 plex (K) were selected as fixed modifications, while Oxidation (M), TMT 6/10/16 plex (Y) and phospho (S/T/Y) was added as variable modifications. The FDR for phosphopeptides was set to 0.01. Searches were performed against the Gallus gallus UniProt FASTA database (February 2020) containing 34 878 entries. Quantification of phosphopeptides was normalized by subtracting the median intensity of each sample. Phosphopeptides that changed by > 1.5 fold with a Student t-test *p* < 0.05 were considered significantly regulated.

With regard to the kinase–substrate enrichment analysis, iGPS [58] was used to identify any phosphosite on a kinase. GSEA version 4.1.0 [59] was used to identify significantly enriched kinases using a ranked fold change of all quantified phosphopeptides. The corresponding p value and normalized enrichment score were assigned for each kinase.

### Targeted metabolomics

Targeted metabolite profiling was performed using liquid chromatography–tandem mass spectrometry (LC-MS/MS). Briefly, HILIC separation was achieved on an ACQUITY UPLC BEH Amide column (1.7 μm, 2.1 mm × 100 mm; Waters MS Technologies, Manchester, UK) with the Nexera Ultra High-Performance Liquid Chromatography system (1290 Infinity LC; Agilent Technologies). The gradient established by mobile phase A (15 mM CH_3_COONH_4_ in HPLC water) and mobile phase B (acetonitrile) was set as follows: 0−18 minutes, 90%−40% B; 18−18.1 minutes, 40%−90% B; 18.1−18.2 minutes, 90%−85% B; and 18.2−23 minutes, 85% B. Target compounds were detected using an AB QTRAP 5500 (SCIEX, Foster City, CA, USA) mass spectrometer with negative ion electrospray ionization in the multiple reaction monitoring mode. The multiple reaction monitoring mode conditions were set as follows: source temperature of 450°C and ion source gas 1, 45 psi; ion source gas 2, 45 psi; curtain gas, 30 psi; and ion spray voltage floating, −4500 V. The chromatographic peak area and retention time were extracted using MultiQuant software (SCIEX). Quality control samples, which were prepared from pooled standard substances for the targeted metabolites, were loaded at regular intervals in the analysis sequence (one quality control after every 5 samples) to monitor the precision and stability of the system. Differential metabolites were identified on the basis of a variable importance projection >1.0 obtained from the OPLS-DA analysis and *p* < 0.05 from Student’s t-test.

### Isolation and culture of cESCs

Fertile fresh laid eggs (EG&K stage X) were collected to isolate cESCs according to the method of Aubel and Pain [60] with some modifications. In brief, eggs were disinfected with 75% ethanol and then broken to isolate the blastoderm using sterile dissecting scissors and spoons. Isolated blastoderms were immersed in PBS and washed gently to remove the excess yolk. Cleaned blastoderms were then immersed in new PBS and dissociated mechanically by repeated up and down gentle pipetting. Cell suspensions were then filtered through a 200-mesh sieve, followed by low-speed centrifugation. Collapsed cells were resuspended in Knockout-DMEM (Cat: 10829018; Gibco, Grand Island, NY, USA), supplemented with 10% fetal bovine serum ([FBS] Gibco), 2% chicken serum (Cat: 16110-082; Gibco), 0.4% non-essential amino acids (Cat: M7145; Sigma), 2 mmol/L, L-glutamine (Invitrogen, Carlsbad, CA, USA), 1 mmol/L, β-mercaptoethanol, 100 U/ml penicillin (Gibco), 100 μg/ml streptomycin (Gibco) and recombinant cell factors. These factors included 1 ng/ml LIF (Cat: 8878-LF; R&D Systems, Minneapolis, MN, USA), 10 ng/ml mSCF (Cat: 7466-SC; R&D Systems) and 10 ng/ml bFGF (Cat: 233-FB; R&D Systems). Cells were cultured in 12-well plates at 38.5°C under a water-saturated atmosphere containing 95% air humidity and 5% CO_2_. The cESCs were stimulated with one or more of the following: 50 ng/ml TPA (Cat: sc-3576; SantaCruz, CA, USA), 10 μg/ml Go:6983 (Cat: HY-13689; MCE, Monmouth Junction, NJ, USA) and 10 μg/ml BAY 11-7082 (Cat: sc-200615; SantaCruz). Cells were stimulated for 2 hours unless otherwise stated. The chicken fibroblast cell line DF-1 was maintained in DMEM/F12 medium with 10% FBS (Gibco), 100 mg/ml streptomycin and 100 U/ml penicillin at 37°C with 5% CO_2_ in a humidified incubator.

### IRF1 knockdown and overexpression

To overexpress IRF1, the full-length chicken IRF1 coding sequence (NM_205415.2) was inserted into a pHBLV-U6-MCS-CMV-ZsGreen-PGK-PURO expression vector. To knockdown IRF1 expression, three shRNAs against IRF1 and a control shRNA were designed (shRNA1: 5′-GCGCCATGAATTCACTGCCTGACAT-3′, shRNA2: 5′-CCACCTCTGACAAAGGACCAGAAGA-3′, shRNA3: 5′-AAGGAAGTCAAAGTCTTCAAGGGAA-3′ and non-specific control shRNA: 5′-TTCTCCGAACGTGTCACGTAA-3′), and cloned into the same expression vector. The constructed vectors were transfected into HEK293T cells together with psPAX2 and pMD2G vectors. Two days after the transfection, viruses were collected and precipitated by ultracentrifugation. The viruses were suspended in PBS, and aliquoted and stored at −80°C. Obtained lentivirus were transfected into 293T cells to determine the viral titer by the method of end point dilution through counting the numbers of ZsGreen-positive cells under a fluorescence microscope. The titers of lentivirus used for cultured cells were > 1 × 10^8^ TU/ml, while those used for egg injection were > 1 × 10^9^ TU/ml. Lentivirus containing empty vectors and lentiviruses containing non-specific shRNA served as controls.

### Immunofluorescence, nuclear staining and imaging

For analysis of the chicken blastoderm, at least 10 blastoderms were pooled together and centrifuged at 2000 × g for 5 minutes. The resulting tissue masses were paraffin-embedded, and then 4-μm thick sections were cut and placed on slides. Tissue sections or cell slides were fixed in cold methanol and acetone (1:1 mixture) for 2 hours, washed with PBS and permeabilized with 0.1% Triton X-100 in PBS for 15 minutes. This was followed by blocking using 10% donkey or goat FBS for 30 minutes at 37°C. The sections were incubated overnight at 4°C with primary antibodies against H3S10P (Cat: 3377T; CST; 1:200), PCNA (Cat: abs120180; Absin, Shanghai, China; 1:200), PKC (Cat: NB600-201; Novus, Littleton, CO, USA; 1:100) or P65 (Cat: NBP2-24541; Novus; 1:100). The slides were washed with PBS and then incubated with FITC-conjugated or PE-conjugated secondary antibodies (Abcam) for 2 hours at room temperature. The cell nuclei were counterstained with DAPI (Invitrogen) for 15 minutes at room temperature. The slides were then examined by laser scanning confocal microscopy (Nikon A1HD25; Nikon Corporation, Tokyo, Japan).

### Flow cytometric cell cycle analysis

Cultured cells were harvested and washed with PBS to analyze the cell cycle. The collected cells were fixed with 75% alcohol overnight at 4°C and then treated with RNase I (Sigma) for 15 minutes. After washing with PBS, the cells were stained with 50 mg/ml propidium iodide (Sigma). The cells were then immediately subjected to LSRII flow cytometry (Beckman Coulter, Inc., Brea, CA) and analyzed using FlowJo v10.0.8. A minimum of 20 000 cells were analyzed for each sample.

### EdU incorporation assay

EdU incorporation was evaluated by the TransDetect® EdU 488 Fluorophore Imaging Kit according to the manufacturer’s instructions (TransDetect, Beijing, China). Briefly, cells were cultured in medium containing 20 μM EdU for 2 hours. After removal of the medium, the cells were washed, fixed in methanol for 20 minutes and permeated with PBS containing 0.5% Triton X-100. EdU incorporation was detected by Click-it chemistry with Alexa Fluor 488 according to the manufacturer’s instructions. The cell nuclei were counterstained with DAPI (Invitrogen) for 15 minutes, and the fluorescence signals were visualized under a confocal microscope (Nikon A1HD25).

### PKC activity and NF-κB transcriptional activity

PKC activity in cell lysates was assayed using the PKC Kinase Activity Assay Kit (Cat: ab139437; Abcam, Cambridge, MA, USA) according to the manufacturer’s instructions. NF-κB transcriptional activity was determined using the pGL4 luciferase reporter vector, which contains five copies of NF-κB response elements. The cells were transfected with plasmid DNA using Lipofectamine^TM^ 3000 (Invitrogen) transfection reagent. Cell lysates were collected, and luciferase activity was evaluated using the Bright-Glo™ luciferase detection assay according to the manufacturer’s protocol (Cat: E2610; Promega, Madison, WI, USA). Luciferase was measured using the Infinite 200 Microplate Reader (Tecan, Mnnedorf, Switzerland).

### Egg windowing and injection

A total of 140 fertilized eggs were randomly divided into two groups, each containing 70 eggs. All eggs were swabbed with 70% ethanol and then laid flat for 1 hour. A window of approximately 0.5 cm in diameter was cut in the equatorial plane of the eggshell to expose the blastoderm. A volume of 2 μL of recombinant lentivirus expressing IRF1 or control empty lentivirus wase injected into the subgerminal cavity of the embryo using a microinjector (Tritech Research, Los Angeles, CA, USA). Micropipettes of 40 μm in diameter, used for injection, were made from borosilicate glass capillaries drawn out with a micropipette puller. After the injection, windows were sealed with adhesive tape. Eggs were then incubated in a specialized incubator maintained at 37.8°C and 75% relative humidity.

### Statistical analysis

All data are presented as the mean ± SEM unless otherwise indicated. Statistical analyses were carried out by student t-test using SPSS version 20.0. A *p* value < 0.05 was considered as significant. Graphics were drawn using GraphPad Prism software (version 8.0.1.), *p < 0.05; **p < 0.01; ***p < 0.001 were labeled.

## Supplementary Information

**Additional file 1: Fig. S1.** Complementary data shows the differences between pre-diapause and diapause embryos. (A), Immunostaining of blastoderm sections with an antibody against the proliferation marker PCNA. Samples were counterstained with DAPI to visualize DNA (blue). Scale bar, 40 μm. (B) Differences in expression between blastoderms isolated from fresh oviposited eggs (F0) or stored at low temperature for 8 days (S8d) of genes associated with cell proliferation, N = 4. (C), TUNEL assay of blastocysts (green). Samples were counterstained with DAPI to visualize DNA (blue). Scale bar, 40 μm. (D), Box plots showing the abundance of representative metabolites in F0 or S8d blastoderm. (E), Differences in expression between blastoderms isolated from F0 and S8d of genes encoding key enzyme in the TCA cycle, N = 4. **Fig. S2.** Data summary of the RNA-Seq data generated during the initiation and maintaining of diapause. (A), Evaluation of RNA-Seq data generated from blastoderms isolated from fresh oviposited or low temperature-stored eggs. (B), Venn diagram showing the number of DEGs identified by RNA-Seq. (C, D), RNA-Seq expression levels of proliferation associated genes (C), and pluripotency markers genes (D), represented as heatmaps. Genes involved in negative regulation proliferation are marked in red while genes involved in negative regulation proliferation are marked in blue. **Fig. S3.** Data summary of the RNA-Seq data generated during the termination of diapause. (A), Evaluation of RNA-Seq data generated from blastoderms isolated from incubated eggs. (B), Scatterplot comparing the fold change of gene expression during the reactivation process. Each dot represents a gene quantified by RNA-Seq. **Fig. S4.** Complementary data for Fig. 4E shows the expressions and functions of the screened genes. (A), RNA-Seq data showing dynamic changes in the expression of genes at all time points. (B), DAVID analysis of enriched GO terms for the 19 screened genes. (C), Protein-protein interaction network of the screened genes. The line thickness indicates the strength of data support. (D), qPCR of changes in gene expression after ESCs were induced by cold stress (28°C) for 3 hours (C3h) or 6 hours (C6h), N = 5. **Fig. S5.** Data summary of the phosphoproteomic data. (A), Heatmap showing the Pearson correlation coefficients between the different biological replicates. (B), Principal component analysis of phosphoproteomic data. (C), Distribution of serine (S), threonine (T) and tyrosine (Y) phosphorylation sites. (D), Number of significantly regulated phosphopeptides identified by different comparations. The threshold was set as P value < 0.05 and fold change >1.5. (E), Heatmap shows the phosphorylation levels of cell cycle associated proteins. (F), Histogram of phosphorylation levels of the indicated phosphosites in Marcks and Marcksl1 peoteins. **Fig. S6.** mTOR pathways is not significantly changed during the transition between pre-diapause and diapause. (A), RNA-Seq expression levels of mTOR related genes. The size and color show the average RNA-Seq normalized expression values across replicates. (B), Phosphoproteomics data shows the phosphorylation level of mTOR target protein sites. Data was represented as heatmap, color show the normalized phosphoproteomics values. (C), Western blot analysis of the pre-diapause and diapause embryos with ribosome protein S6 (Cat: 2217S; CST) and its phosphorylated form pS6 (Cat: 2211S; CST) antibody. GAPDH was used as used as the loading control. **Fig. S7.** Changes in gene expression after ESCs were stimuli by cold stress (28°C) for 3 hours (C3h) or 6 hours (C6h), N = 5. **Fig. S8.** Complementary data for Fig. 7 shows effects of OE-IRF1 on cell cycle and embryonic growth. (A), Representative flow cytometry histogram showing cell cycle analysis. (B), Statistics of cell cycle results from (B) based on three technical replicates. (C), Verification of the efficacy of lentiviral mediated IRF1 overexpression (OE-IRF1) in blastoderms, N = 5. (D), Changes in diameter of the blastoderms after IRF1 overexpression, N = 6. For all figures, mean ± SEM is shown. *p <0.05; **p < 0.01; ***p <0.001.

## Data Availability

RNA-Seq data were deposited in SRA under accession PRJNA778435 [61]. Phosphoproteomic data were deposited in the ProteomeXchange database under the accession PXD029941 [62].

## Abbreviations

OPLS-DA: Orthogonal partial least-squares discriminant analysis
VIP: Variable importance projection
PCA: Principal component analysis
FDR: False discovery rate
DEGs: Differentially expressed genes
cESCs: Chicken embryonic stem cells
TPA: 12-O-tetradecanoylphorbol13-acetate
EdU: 5-Ethynyl-2′-deoxyuridine
OE-IRF1: Overexpression of IRF1
SCX: Strong cation exchange
FBS: Fetal bovine serum
