# Supplementary material for: Temperature-induced embryonic diapause in chickens is mediated by PKC-NF-κB-IRF1 signaling
Source: BMC Biol. 2023 Mar 8;21:52. doi: 10.1186/s12915-023-01550-0 (PMC9993608; doi:10.1186/s12915-023-01550-0)
Supplement: Supplementary file 1 — Additional file 1: Fig. S1. Complementary data shows the differences between pre-diapause and diapause embryos. (A), Immunostaining of blastoderm sections with an antibody against the proliferation marker PCNA. Samples were counterstained with DAPI to visualize DNA (blue). Scale bar, 40 μm. (B) Differences in expression between blastoderms isolated from fresh oviposited eggs (F0) or stored at low temperature for 8 days (S8d) of genes associated with cell proliferation, N = 4. (C), TUNEL assay of blastocysts (green). Samples were counterstained with DAPI to visualize DNA (blue). Scale bar, 40 μm. (D), Box plots showing the abundance of representative metabolites in F0 or S8d blastoderm. (E), Differences in expression between blastoderms isolated from F0 and S8d of genes encoding key enzyme in the TCA cycle, N = 4. Fig. S2. Data summary of the RNA-Seq data generated during the initiation and maintaining of diapause. (A), Evaluation of RNA-Seq data generated from blastoderms isolated from fresh oviposited or low temperature-stored eggs. (B), Venn diagram showing the number of DEGs identified by RNA-Seq. (C, D), RNA-Seq expression levels of proliferation associated genes (C), and pluripotency markers genes (D), represented as heatmaps. Genes involved in negative regulation proliferation are marked in red while genes involved in negative regulation proliferation are marked in blue. Fig. S3. Data summary of the RNA-Seq data generated during the termination of diapause. (A), Evaluation of RNA-Seq data generated from blastoderms isolated from incubated eggs. (B), Scatterplot comparing the fold change of gene expression during the reactivation process. Each dot represents a gene quantified by RNA-Seq. Fig. S4. Complementary data for Fig. 4E shows the expressions and functions of the screened genes. (A), RNA-Seq data showing dynamic changes in the expression of genes at all time points. (B), DAVID analysis of enriched GO terms for the 19 screened genes. (C), Protein-protein i [file 12915_2023_1550_MOESM1_ESM.pdf]

Fig. S1

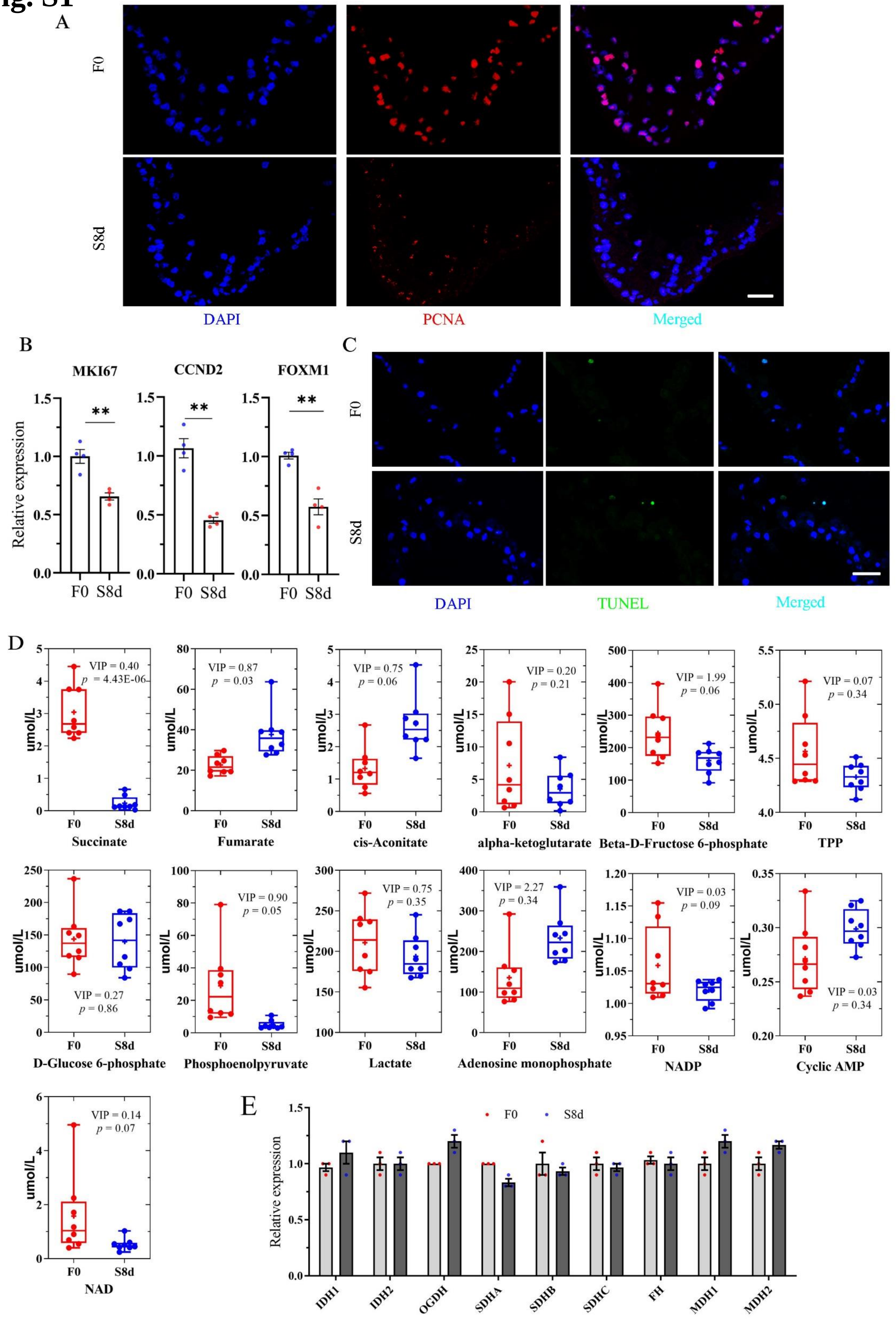
A
F0
S8d
DAPI
PCNA
Merged
B
MKI67
CCND2
FOXM1
Relative expression
F0 S8d
C
DAPI
TUNEL
Merged
D
umol/L
Succinate
VIP = 0.40
p = 4.43E-06
Fumarate
VIP = 0.87
p = 0.03
cis-Aconitate
VIP = 0.75
p = 0.06
alpha-ketoglutarate
VIP = 0.20
p = 0.21
Beta-D-Fructose 6-phosphate
VIP = 1.99
p = 0.06
TPP
VIP = 0.07
p = 0.34
D-Glucose 6-phosphate
VIP = 0.27
p = 0.86
Phosphoenolpyruvate
VIP = 0.90
p = 0.05
Lactate
VIP = 0.75
p = 0.35
Adenosine monophosphate
VIP = 2.27
p = 0.34
NADP
VIP = 0.03
p = 0.09
Cyclic AMP
VIP = 0.03
p = 0.34
NAD
VIP = 0.14
p = 0.07
E
F0
S8d
Relative expression
IDH1
IDH2
OGDH
SDHA
SDHB
SDHC
FH
MDH1
MDH2

Fig. S2

A

Samples	Clean reads	%≥Q30	Mapped reads	Mapped ratio
F0-1	62,464,006	93.94%	57,599,583	92.21%
F0-2	61,464,570	94.12%	56,632,643	92.14%
F0-3	60,896,134	93.95%	56,180,728	92.26%
S1d-1	111,126,782	93.91%	101,397,856	91.25%
S1d-2	44,641,682	92.95%	41,146,386	92.17%
S1d-3	51,368,560	93.34%	46,405,407	90.34%
S2d-1	48,212,782	91.99%	44,140,407	91.55%
S2d-2	41,910,602	92.82%	38,611,869	92.13%
S2d-3	43,531,524	92.32%	39,931,738	91.73%
S4d-1	42,424,074	92.06%	38,759,705	91.36%
S4d-2	43,414,560	91.78%	39,730,506	91.51%
S4d-3	42,031,684	92.25%	38,471,342	91.53%
S8d-1	47,965,750	92.41%	43,948,539	91.62%
S8d-2	40,808,366	92.00%	37,371,562	91.58%
S8d-3	40,312,962	92.89%	37,376,004	92.71%
S12d-1	44,460,670	92.63%	40,937,201	92.08%
S12d-2	42,837,182	92.33%	39,378,118	91.93%
S12d-3	41,601,818	92.88%	38,362,996	92.21%

B

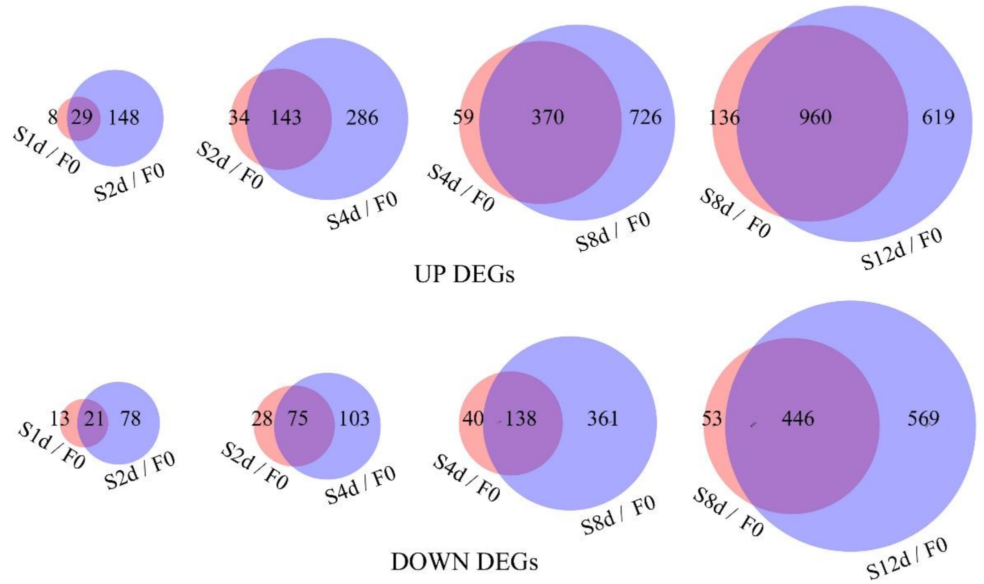

C

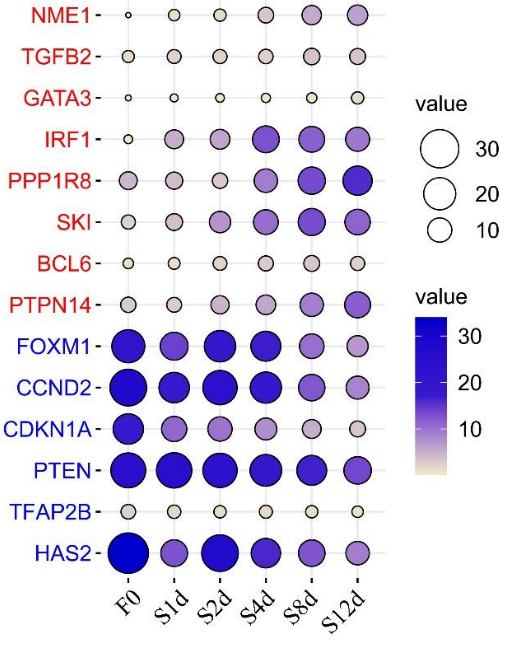

D

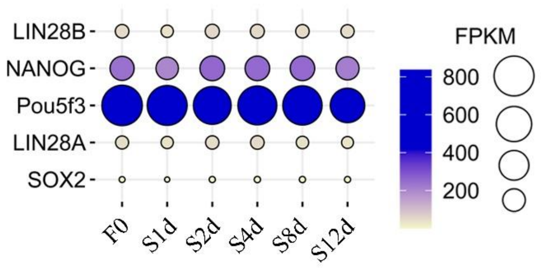

Fig. S3

A

Samples	Clean reads	%≥Q30	Mapped reads	Mapped ratio
In0.5h-1	73,959,434	93.86%	68,114,265	92.10%
In0.5h-2	71,815,394	93.80%	66,218,914	92.21%
In0.5h-3	68,807,322	94.03%	63,502,538	92.29%
In1h-1	45,349,138	94.10%	41,790,948	92.15%
In1h-2	58,687,116	93.30%	54,042,323	92.09%
In1h-3	62,073,986	93.43%	56,987,168	91.81%
In2h-1	52,659,216	93.51%	48,491,896	92.09%
In2h-2	57,444,728	93.97%	53,110,687	92.46%
In2h-3	51,535,706	94.51%	47,720,179	92.60%
In4h-1	52,245,874	93.92%	48,121,248	92.11%
In4h-2	52,185,126	93.89%	48,021,209	92.02%
In4h-3	50,209,184	93.54%	45,866,203	91.35%
In6h-1	55,625,774	94.32%	51,262,523	92.16%
In6h-2	59,256,852	94.16%	54,580,123	92.11%
In6h-3	51,044,372	94.14%	46,983,474	92.04%
In12h-1	62,899,476	94.13%	57,966,890	92.16%
In12h-2	58,003,682	93.66%	53,169,087	91.67%
In12h-3	46,342,960	94.13%	42,709,911	92.16%

B

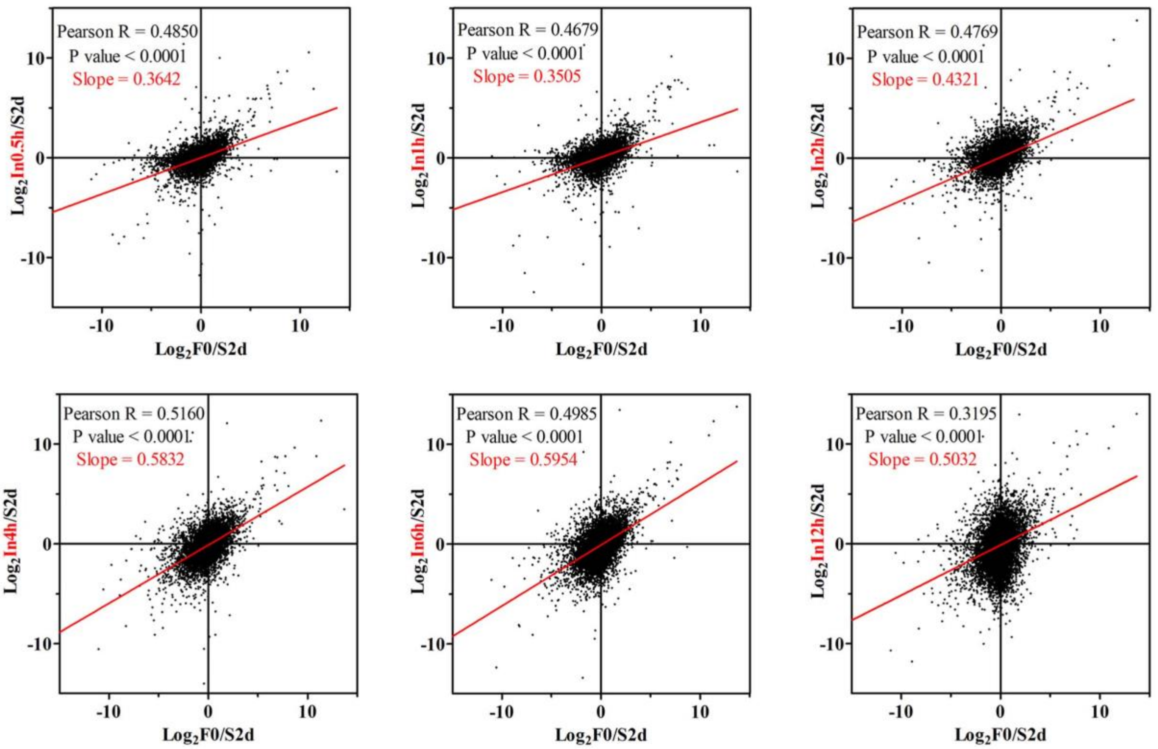

Fig. S4

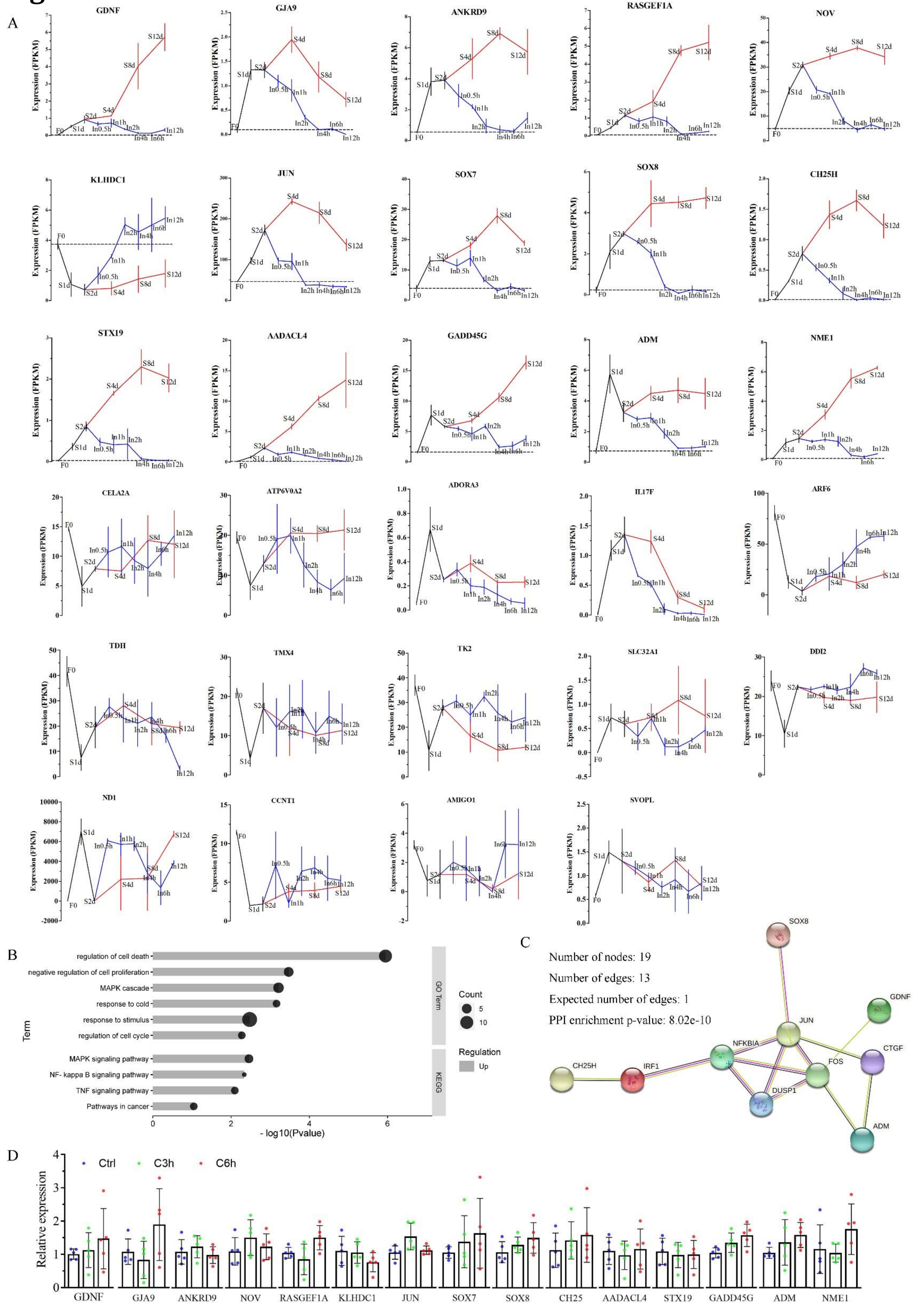
A
GDNF
GJA9
ANKRD9
RASGEF1A
NOV
KLHDC1
JUN
SOX7
SOX8
CH25H
STX19
AADACL4
GADD45G
ADM
NME1
CELA2A
ATP6V0A2
ADORA3
IL17F
ARF6
TDH
TMX4
TK2
SLC32A1
DDI2
ND1
CCNT1
AMIGO1
SVOPL
Expression (FPKM)
F0
S1d
S2d
S4d
S8d
S12d
In0.5h
In1h
In2h
In4h
In6h
In12h
B
regulation of cell death
negative regulation of cell proliferation
MAPK cascade
response to cold
response to stimulus
regulation of cell cycle
MAPK signaling pathway
NF- kappa B signaling pathway
TNF signaling pathway
Pathways in cancer
Term
- log10(Pvalue)
GO Term
KEGG
Count
5
10
Regulation
Up
C
Number of nodes: 19
Number of edges: 13
Expected number of edges: 1
PPI enrichment p-value: 8.02e-10
SOX8
GDNF
JUN
CTGF
NFKBIA
FOS
CH25H
IRF1
DUSP1
ADM
D
Ctrl
C3h
C6h
Relative expression
GDNF
GJA9
ANKRD9
NOV
RASGEF1A
KLHDC1
JUN
SOX7
SOX8
CH25
AADACL4
STX19
GADD45G
ADM
NME1

Fig. S5

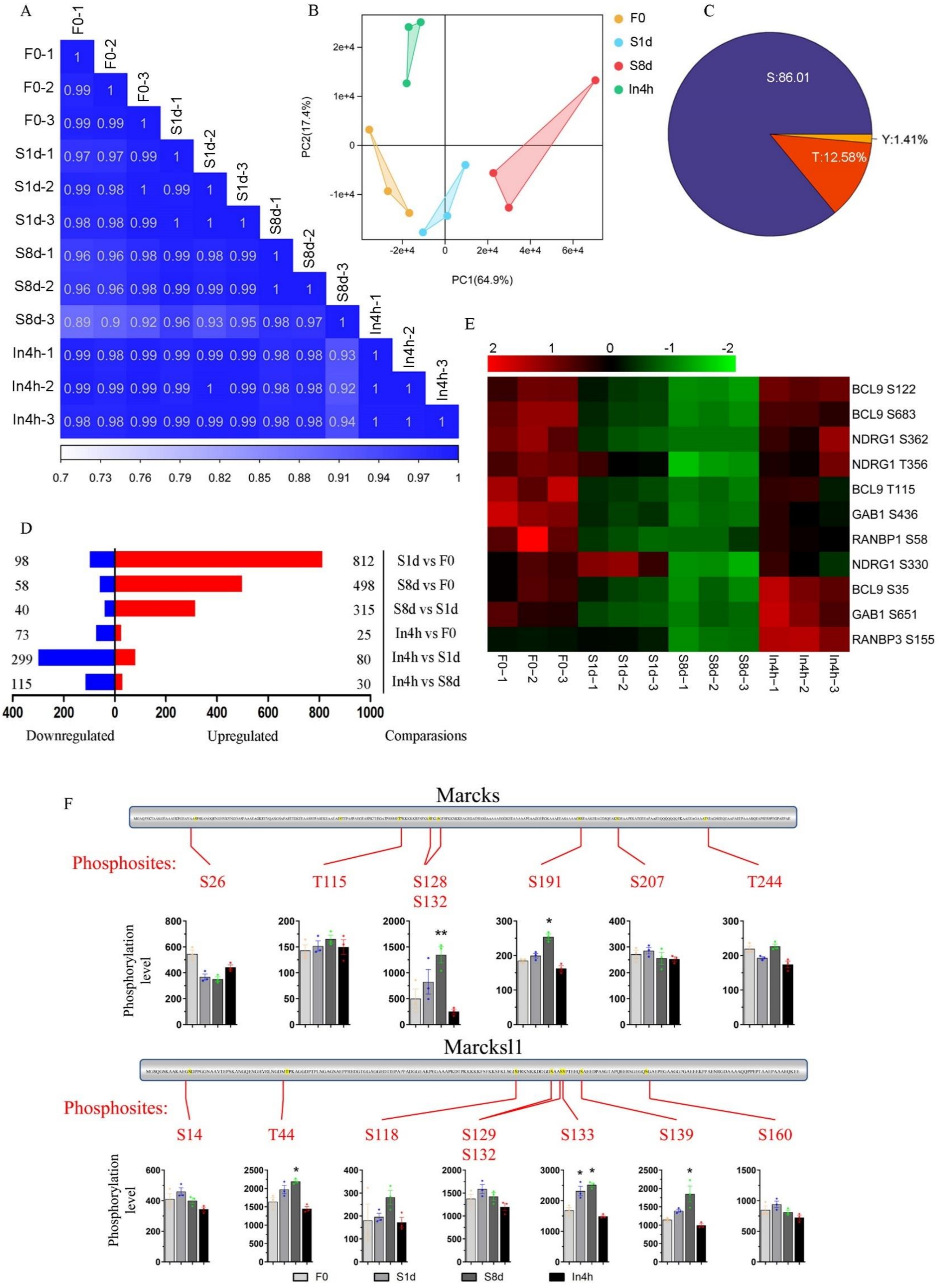
A
F0-1
F0-2
F0-3
S1d-1
S1d-2
S1d-3
S8d-1
S8d-2
S8d-3
In4h-1
In4h-2
In4h-3
0.7 0.73 0.76 0.79 0.82 0.85 0.88 0.91 0.94 0.97 1
B
F0
S1d
S8d
In4h
PC1(64.9%)
PC2(17.4%)
C
S:86.01
Y:1.41%
T:12.58%
D
98 812 S1d vs F0
58 498 S8d vs F0
40 315 S8d vs S1d
73 25 In4h vs F0
299 80 In4h vs S1d
115 30 In4h vs S8d
Downregulated
Upregulated
Comparasions
E
BCL9 S122
BCL9 S683
NDRG1 S362
NDRG1 T356
BCL9 T115
GAB1 S436
RANBP1 S58
NDRG1 S330
BCL9 S35
GAB1 S651
RANBP3 S155
F
Marcks
Phosphosites:
S26
T115
S128
S132
S191
S207
T244
Phosphorylation level
Marcksl1
Phosphosites:
S14
T44
S118
S129
S132
S133
S139
S160
F0
S1d
S8d
In4h

Fig. S6

A

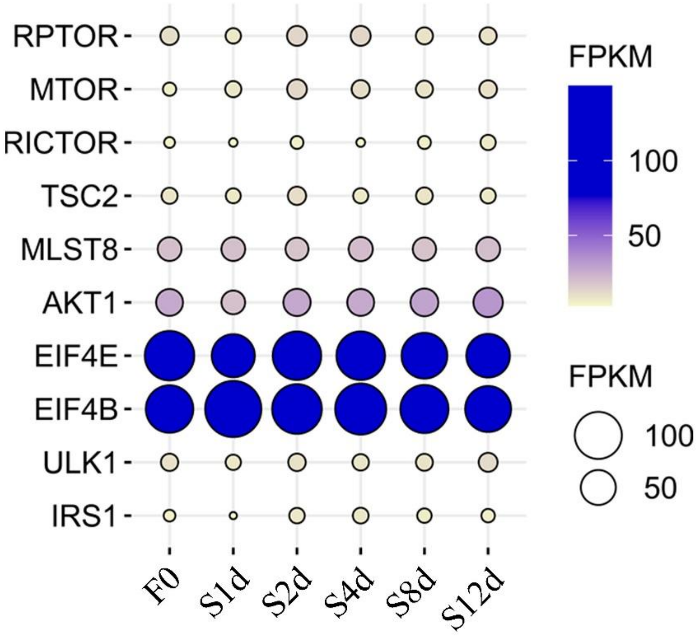
RPTOR
MTOR
RICTOR
TSC2
MLST8
AKT1
EIF4E
EIF4B
ULK1
IRS1
F0
S1d
S2d
S4d
S8d
S12d
FPKM
100
50
FPKM
100
50

B

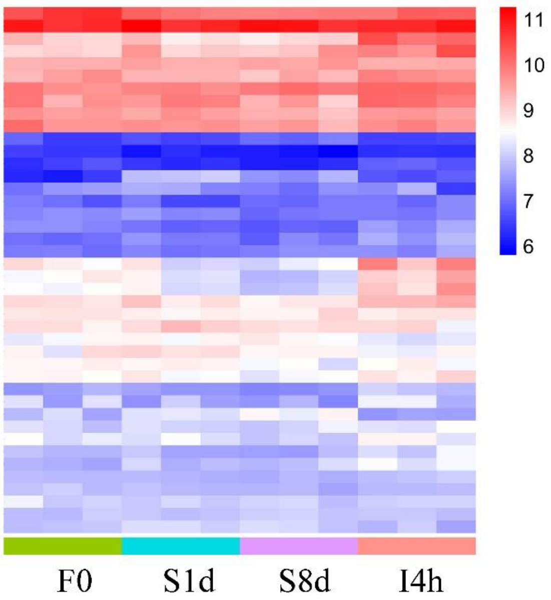
11
10
9
8
7
6
F0
S1d
S8d
I4h

C

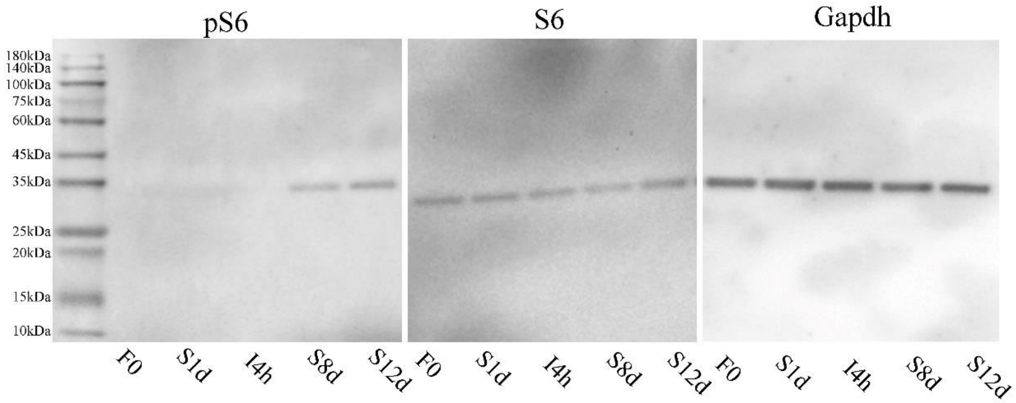
pS6
S6
Gapdh
180kDa
140kDa
100kDa
75kDa
60kDa
45kDa
35kDa
25kDa
20kDa
15kDa
10kDa
F0
S1d
I4h
S8d
S12d
F0
S1d
I4h
S8d
S12d
F0
S1d
I4h
S8d
S12d

Fig. S7

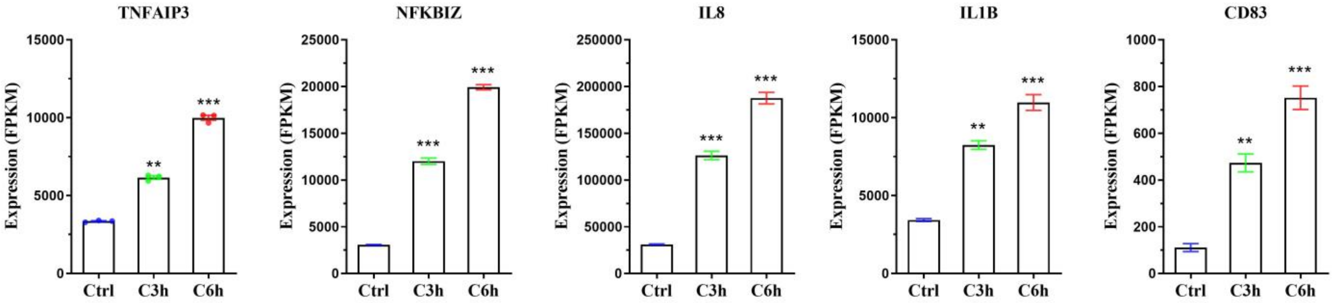
TNFAIP3
NFKBIZ
IL8
IL1B
CD83
Expression (FPKM)
Ctrl
C3h
C6h
**
***

Fig. S8

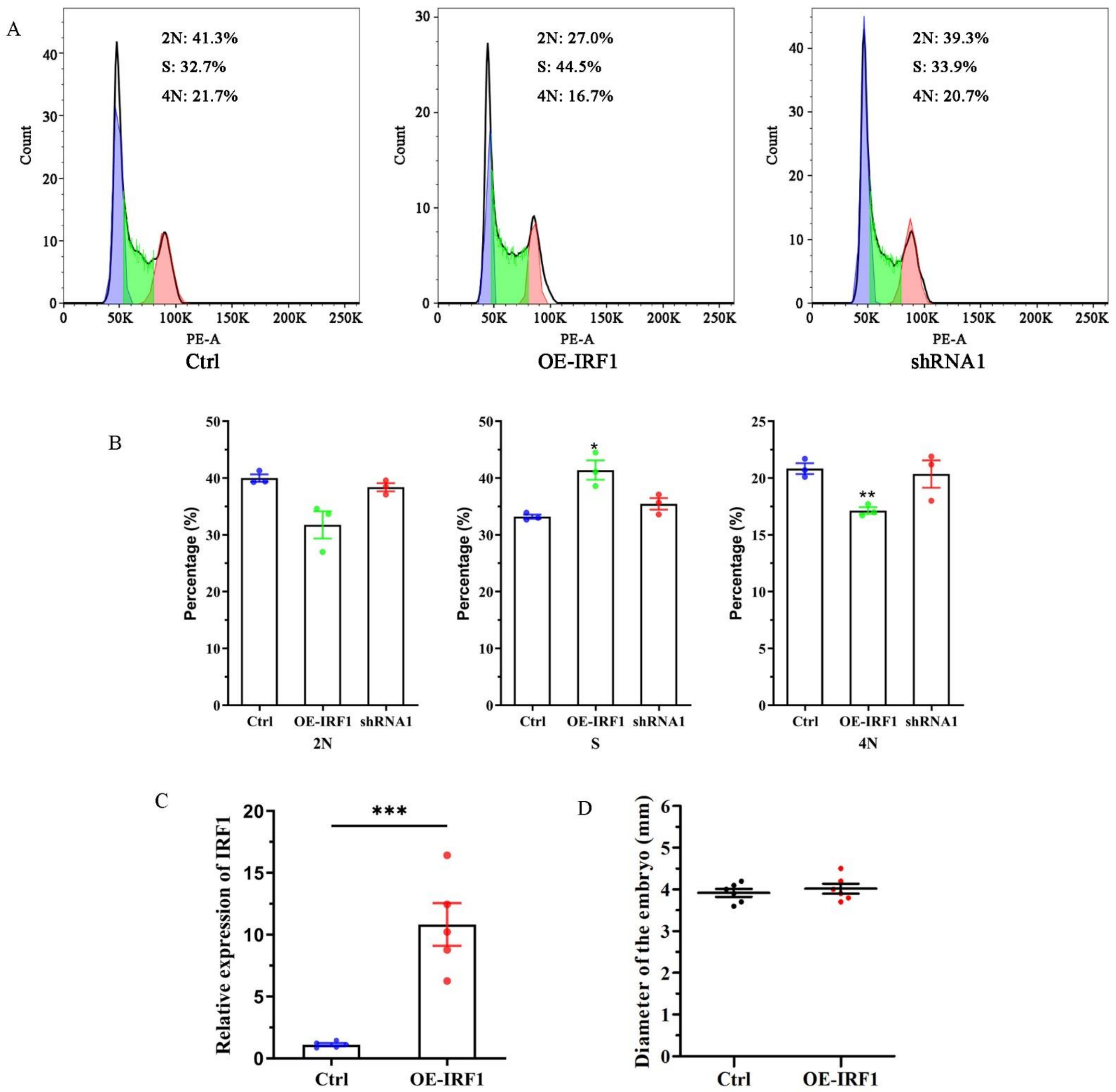
A
2N: 41.3%
S: 32.7%
4N: 21.7%
2N: 27.0%
S: 44.5%
4N: 16.7%
2N: 39.3%
S: 33.9%
4N: 20.7%
Count
PE-A
Ctrl
OE-IRF1
shRNA1
B
Percentage (%)
2N
S
4N
C
Relative expression of IRF1
***
D
Diameter of the embryo (mm)
